# The Limited Incorporation and Role of Fluorine in Mn-rich Disordered Rocksalt Cathodes

**DOI:** 10.1021/acsenergylett.4c01075

**Published:** 2024-05-30

**Authors:** Vincent C. Wu, Peichen Zhong, Julia Ong, Eric Yoshida, Andrew Kwon, Gerbrand Ceder, Raphaële J. Clément

**Affiliations:** † Materials Department and Materials Research Laboratory, University of California—Santa Barbara, Santa Barbara, California 93106, United States; ‡ Department of Materials Science and Engineering, University of California—Berkeley, Berkeley, California 94720, United States; ∥ Materials Sciences Division, Lawrence Berkeley National Laboratory, Berkeley, California 94720, United States

## Abstract

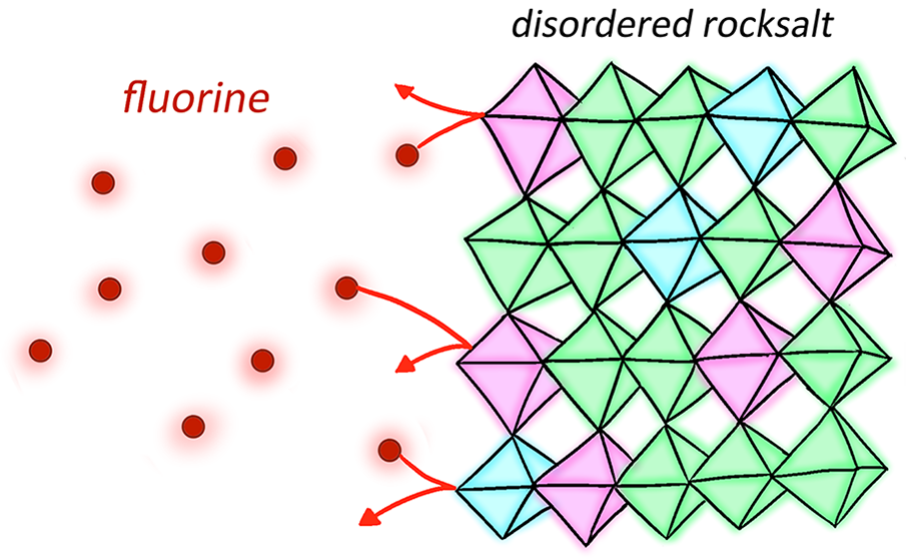

Disordered rocksalt oxide (DRX) cathodes are promising candidates for next-generation Co- and Ni-free Li-ion batteries. While fluorine substitution for oxygen has been explored as an avenue to enhance their performance, the amount of fluorine incorporated into the DRX structure is particularly challenging to quantify and impedes our ability to relate fluorination to electrochemical performance. Herein, an experimental–computational method combining ^7^Li and ^19^F solid-state nuclear magnetic resonance, and *ab initio* cluster expansion Monte Carlo simulations, is developed to determine the composition of DRX oxyfluorides. Using this method, the synthesis of Mn- and Ti-containing DRX via standard high temperature sintering and microwave heating is optimized. Further, the upper fluorination limit attainable using each of these two synthesis routes is established for various Mn-rich DRX compounds. A comparison of their electrochemical performance reveals that the capacity and capacity retention mostly depend on the Mn content, while fluorination plays a secondary role.

The development of high energy density electrode materials for Li-ion batteries is pivotal to the deployment of cost-effective and sustainable energy storage solutions and the transition to renewable energy sources and electric modes of transportation. Significant research efforts have focused on fine-tuning the composition of existing cathode chemistries (e.g., LiCoO_2_) to increase their reversible capacity and capacity retention and enhance their thermal and high voltage stability, leading to the commercialization of NMC (Ni, Mn, Co)- and NCA (Ni, Co, Al)-type layered rocksalt oxide structures.^1−3^ While compositional modulation has largely centered on cationic substitutions, anion site tuning can provide a new handle on the electrochemical properties. In particular, the partial substitution of oxygen (O) by fluorine (F) in cathode materials is often desirable, as stronger metal–fluorine bonds and suppressed anion redox reactions impart greater cycling stability, while fluorination may increase the average redox potential through the inductive effect.^4,5^ However, attempts to fluorinate NMC- and NCA-type cathodes have been unsuccessful and resulted instead in the phase separation of the transition metal (TM) oxide and LiF due to the inability to form high-energy TM–F bonds at the synthesis temperatures required to stabilize the layered rocksalt structure.^6−8^

Lithium excess disordered rocksalt oxide (DRX) cathodes have received increasing attention over the past few years due to their high energy densities, compositional flexibility, and promise for more sustainable electrochemical energy storage involving Fe- and Mn-based redox processes.^9−11^ In contrast to their layered oxide counterparts, significant fluorination has been achieved in DRX compounds, enabled by disorder-induced Li-rich local environments amenable to fluorination.^6,10,12,13^ Many studies have examined the influence of partial F substitution for O on the electrochemical performance of DRX, showing that higher fluorination levels result in a higher and more reversible capacity, and a slower capacity fade.^14−28^ The benefits of F substitution have largely been attributed to the lower valence of F^–^ compared to O^2–^, allowing for a greater fraction of low valent redox-active species, such as Mn^2+^ or Mn^3+^, to occupy the cation sites.^10,29^ In turn, this increased TM-based redox reservoir reduces the dependence on O-based redox processes that lead to greater irreversibilities, oxygen loss, voltage hysteresis,^30,31^ and particle cracking.^21^ Additional benefits of fluorination include more interconnected Li transport pathways through the DRX structure,^32^ and less severe Jahn–Teller distortions due to modulation of the crystal field stabilization energy by the F^–^ anions.^16^

Despite the decisive role of F in the properties of DRX oxyfluorides, studies published to date have been unable to determine the exact amount of F incorporated into the DRX structure. As a result, the upper fluorination limit attainable under specific synthesis conditions remains unclear. This lack of understanding prevents the controlled synthesis of DRX oxyfluorides and severely impedes the establishment of composition–property–performance relationships, particularly since the Li, TM, and F stoichiometries are interdependent yet each species affects the redox, electronic, and ion transport properties in a unique manner. In most studies published to date, the DRX synthesis product was characterized using laboratory or better synchrotron X-ray diffraction (XRD) or neutron diffraction to examine its phase purity, but such tools cannot distinguish O from F and are therefore of limited use when trying to determine the extent of fluorination. In the absence of notable crystalline impurities, these studies assumed that the target DRX composition was experimentally achieved. A comparatively small number of studies have used ^19^F solid-state nuclear magnetic resonance (ssNMR) to directly probe the distribution of F in the sample, and distinguish F species within the DRX structure from those forming (LiF) impurity phases. Unfortunately, ^19^F ssNMR is not quantitative as a fraction of the F in the DRX phase is “NMR-invisible”.^15^ We have recently devised a methodology to estimate the stoichiometry of the DRX cathode and the composition of the sample, that combines XRD and ^7^Li and ^19^F ssNMR, inductively coupled plasma (ICP) and fluoride-ion selective electrode (F-ISE) measurements, and carbonate titration.^33^ This method, while a significant step forward toward the establishment of composition/property relationships for DRX cathodes, can only provide a range of possible F stoichiometries. Building upon this work, we develop herein a hybrid experimental–computational approach that enables the determination of DRX compositions with greater accuracy, leveraging cluster-expansion Monte Carlo (CEMC) simulations to predict the distribution of F local environments in the DRX cathode of interest and assist the interpretation of the ^19^F ssNMR results. We utilize this method to optimize the synthesis of a series of Mn- and Ti-containing DRX compounds via high temperature sintering or microwave heating of powder precursors with the aim of maximizing fluorination of the DRX phase while also minimizing the amount of impurity phases in the sample. Critically, we find that the degree of DRX fluorination is significantly lower than that previously assumed, particularly for compositions containing a high Mn content. Electrochemical testing of well-characterized DRX compositions (with a Mn content ≥ 0.5) indicates that the Mn content is the single most important compositional handle impacting DRX performance. Further, at a fixed F content, increasing the Mn content is beneficial to long-term capacity retention. These findings provide a new compositional design strategy toward next-generation Mn-rich DRX cathodes.

## Determination of DRX Composition

**Figure 1:**
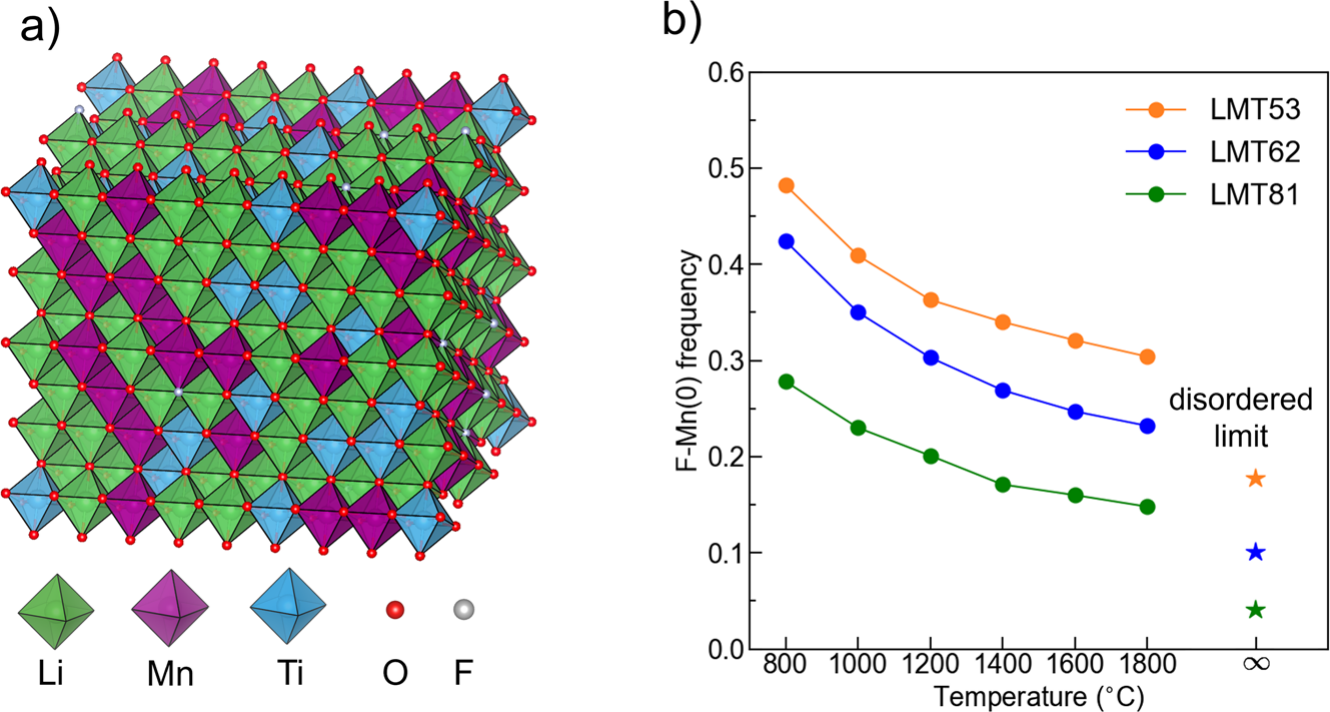
Results from *ab initio* cluster expansion Monte Carlo (CEMC) simulations. (a) Representative DRX supercell generated by a simulation. (b) Fraction of F species with no nearest-neighbor Mn, F–Mn(0), in LMT53, LMT62, and LMT81 DRX compounds at various equilibrium temperatures.

The determination of DRX compositions is complicated by the presence of often partially amorphous impurity phases in as-synthesized DRX powder samples.^33^ While bulk elemental analysis (using ICP and F-ISE measurements) is insufficient to extract the stoichiometry of the DRX phase, quantification via diffraction techniques is difficult due to poorly resolved Bragg peaks. Additionally, the extent of F incorporation into the DRX framework cannot be determined from scattering techniques, as the X-ray and neutron cross sections of O and F are extremely similar. ssNMR provides unique insights into the distribution of ^7^Li and ^19^F environments in a DRX sample and enables the detection of diamagnetic Li- and F-containing impurities.^33^ When combined with bulk elemental analysis, the amount of Li in the DRX phase and in Li-containing impurity phases can be determined quantitatively via ^7^Li ssNMR. In contrast, ^19^F ssNMR is only semiquantitative and underestimates the amount of F in the DRX structure—F species directly bonded to a paramagnetic transition metal ion (here, Mn) in the DRX phase exhibit an extremely rapid decay of their ^19^F ssNMR signal, rendering such F environments effectively invisible by NMR.^33^ The probability of forming “NMR invisible” F environments can be computed assuming a random distribution of species in the rocksalt structure,^33^ although it has been established that some degree of short-range order is always present in as-synthesized DRX,^34^ whereby low energy F–Li bonds are strongly preferred over high energy F–Mn bonds, and this method only provides a lower and an upper bound for the F content. A narrow range of possible F stoichiometries is obtained when the impurity content and/or the amount of Mn in the DRX phase is low. However, when both impurity and Mn contents are high, a wider range is obtained, and this approach becomes less useful. As the uncertainty in the DRX F content stems from an incomplete understanding of the distribution of F environments in the DRX structure, we herein address this issue by simulating this distribution using large DRX supercells and *ab initio* cluster expansion Monte Carlo (CEMC) methods. The CEMC approaches have proven effective at capturing short-range order in multicomponent systems,^35−37^ where a complete thermodynamic model requires consideration of coupled disorder on the cation and anion sublattices.^38,39^ In this work, the CEMC simulations were carried out on a series of Mn^3+^- and Ti^4+^-based DRX oxyfluoride compounds—Li_1.2_Mn_0.5_Ti_0.3_O_1.9_F_0.1_ (LMT53), Li_1.2_Mn_0.6_Ti_0.2_O_1.8_F_0.2_ (LMT62), and Li_1.1_Mn_0.8_Ti_0.1_O_1.9_F_0.1_ (LMT81)—and over a range of temperatures from 800 to 1800 °C to sample the structures from the equilibrium ensemble (Figure 1a) and to determine the equilibrium distribution of F environments at representative synthesis temperatures. The results allow us to derive the fraction of the ^19^F ssNMR signal intensity loss due to the formation of Mn–F bonds and estimate the F content in the DRX phase with unprecedented accuracy. The methodology is described in more detail in Supporting Information Note 1, and the scaling factor used to determine the F content in the DRX phase from the ^19^F ssNMR data is derived in Supporting Information Note 2. A discussion of additional compositions considered for the CEMC simulations can be found in Supplementary Note 3.

The fraction of F environments with no nearest-neighbor Mn derived from the simulations, F–Mn(0), is plotted as a function of the temperature in Figure 1b. F–Mn(0) is the fraction of F environments in the DRX that *can* be observed by NMR. As expected, short-range ordering is more extensive at lower temperatures due to the strong bonding preference between F and Li, resulting in a higher frequency of F–Mn(0) sites, while compositions with a greater Mn content have a lower F–Mn(0) frequency. As the temperature is increased, the configurational entropy contribution to the total energy increases and the equilibrium distribution of cations and anions among available sites in the rocksalt structure becomes more disordered, such that F–Mn(0) decreases and slowly approaches its asymptotic (infinite temperature) value when the distribution of species is fully random. Figure S5 shows that above 800 °C a roughly linear decrease in F–Mn(0) can be observed with increasing Mn content.

### Fluorine Incorporation in Mn^3+^- and Ti^4+^-Based DRX

We investigated the impact of composition and synthesis procedure on the amount of fluorine incorporated into three Mn^3+^- and Ti^4+^-based DRXs. The Mn content was gradually increased across the compositional series, from LMT53 to LMT62 to LMT81, while the Ti and F contents were adjusted to maintain charge balance. All DRXs were prepared via a standard solid-state synthesis and a rapid microwave synthesis, with details of the synthesis procedures discussed in the Methods Section. The latter synthesis route was presented in our recent work^40^ and provides a unique kinetic and thermodynamic pathway toward DRX phase formation, whereby rapid microwave heating to temperatures as high as 1500–1600 °C is followed by a pellet quench. The resulting temperature profile differs substantially from the one obtained when using a long calcination step followed by a slower cooling step, where the pellet is allowed to naturally equilibrate to room temperature, as is typical for solid-state syntheses of DRX cathodes, and may lead to differences in the extent of short-range order and of F incorporation into the DRX structure. A total of six samples were studied: ss-LMT53, ss-LMT63, ss-LMT81, mw-LMT53, mw-LMT62, and mw-LMT81, corresponding to samples prepared via standard solid-state (ss) and microwave (mw) synthesis methods. While the solid-state synthesis of all three compositions has been reported,^20,26,41^ the lack of a comprehensive analytical framework able to determine the purity and composition of the resulting product powders has so far prevented the optimization of the synthesis conditions. Here, we employed the hybrid experimental–computational methodology described earlier to identify the optimal reaction parameters to (1) maximize the F content in the DRX phase and (2) reduce the amount of impurity phases in the as-prepared LMT53, LMT62, and LMT81 samples. For standard solid-state synthesis, we tested various synthesis temperatures from 750 to 1100 °C and calcination times of 2 and 12 h, all under an argon atmosphere. Our results, presented in Figures S6 and S7, show that holding the pellet at the minimum calcination temperature, requiring obtaining a DRX without any layered or spinel competing phase (this temperature increases with Mn content and is equal to 800 °C, 900 °C, and 1000 °C for LMT53, LMT62, and LMT81, respectively), for 12 h maximizes fluorination and reduces the amount of impurities in the sample by minimizing F volatility while allowing sufficient time for F to integrate the DRX structure. For microwave synthesis under air, the microwave heating time was fixed to 5 min, and the microwave power was varied between 600 and 1200 W. Our results, presented in Figures S8 and S9, indicate that lower microwave powers (720 W for LMT53 and 600 W for LMT62 and LMT81) maximize fluorination while also ensuring a sufficiently phase pure DRX sample. Additional details on the optimization of the reaction conditions can be found in Supplementary Note 4.

**Figure 2:**
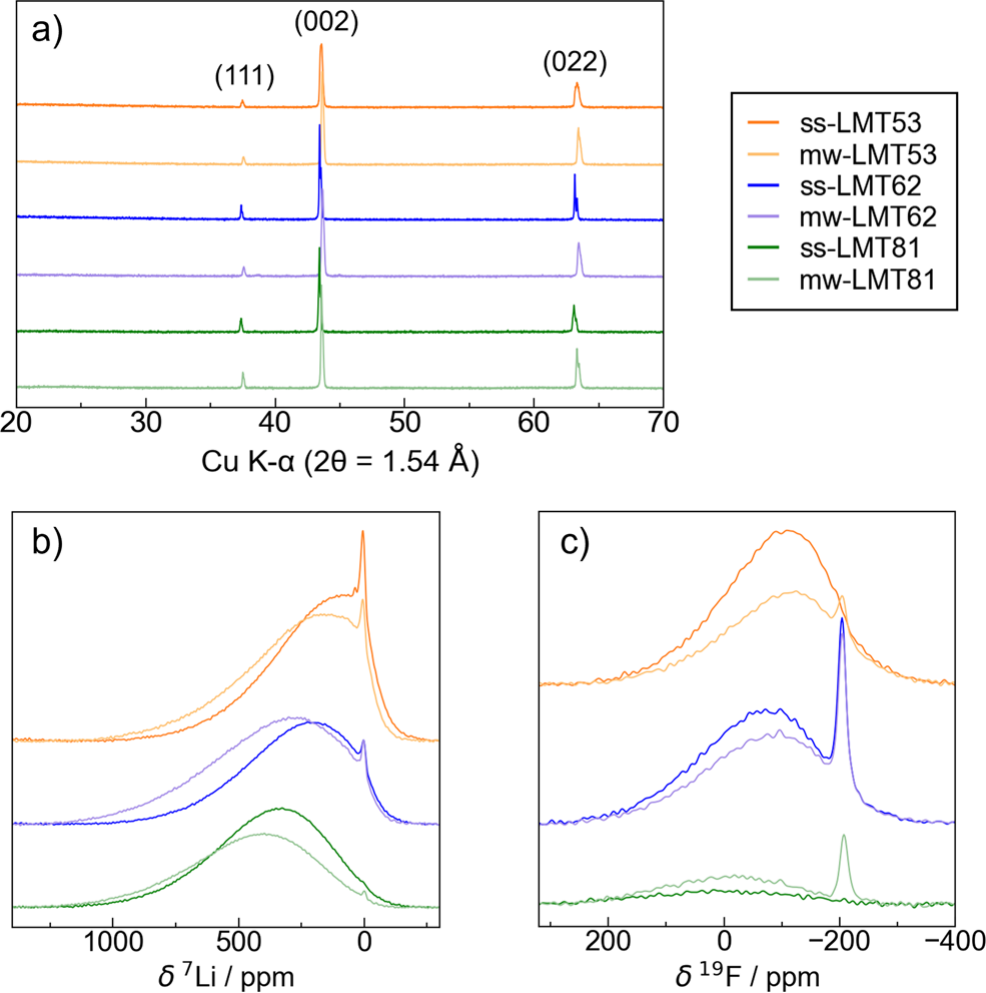
(a) Powder XRD patterns and (b) ^7^Li and (c) ^19^F ssNMR spin echo spectra collected on as-prepared LMT53, LMT62, and LMT81 samples obtained via solid-state and microwave synthesis after optimization of the synthesis parameters: all solid-state syntheses used a 12 h calcination step at 800 °C for LMT53, 900 °C for LMT62, and 1000 °C for LMT81, and all microwave syntheses used a 5 min heating step at 720 W for LMT53 and 600 W for LMT62 and LMT81. ssNMR spectra were acquired at 2.35 T with a magic angle spinning (MAS) speed of 60 kHz with a 50 ms recycle delay and scaled according to the number of scans and the sample mass.

XRD patterns collected on as-prepared DRX powders obtained via solid-state and microwave synthesis using optimized parameters are shown in Figure 2a and show excellent phase purity. A steady shift of the Bragg peaks toward lower angles is observed from LMT53 to LMT62 to LMT81, indicating an expansion of the lattice parameters consistent with the larger ionic radius of Mn^3+^ (0.645 Å) compared to Ti^4+^ (0.605 Å).^42^ For a given DRX composition, the Bragg peaks observed for the microwave synthesized DRX sample are consistently at higher angles compared to those observed for the solid-state synthesized sample, indicating a contraction of the lattice in the former sample, which is attributed to the slight oxidation of Mn^3+^ species to Mn^4+^ (ionic radius of 0.53 Å) upon heating under air.^42^^7^Li and ^19^F ssNMR spectra, shown in Figure 2b,c, reveal a steady shift of the broad paramagnetic NMR line shape attributed to Li/F species within the DRX phase to higher parts per million values with increasing Mn content, as expected from the increased number of paramagnetic interactions between the ^7^Li or ^19^F nuclei and nearby paramagnetic Mn ions.^15,41^ Additionally, for almost all compositions, a sharp diamagnetic signal is present in both ^7^Li and ^19^F ssNMR spectra, attributed to Li- and F-containing impurity phases in the sample, respectively. Fits of sufficiently relaxed ssNMR spectra used for stoichiometric determination are shown in Figures S2–S4.

**Figure 3:**
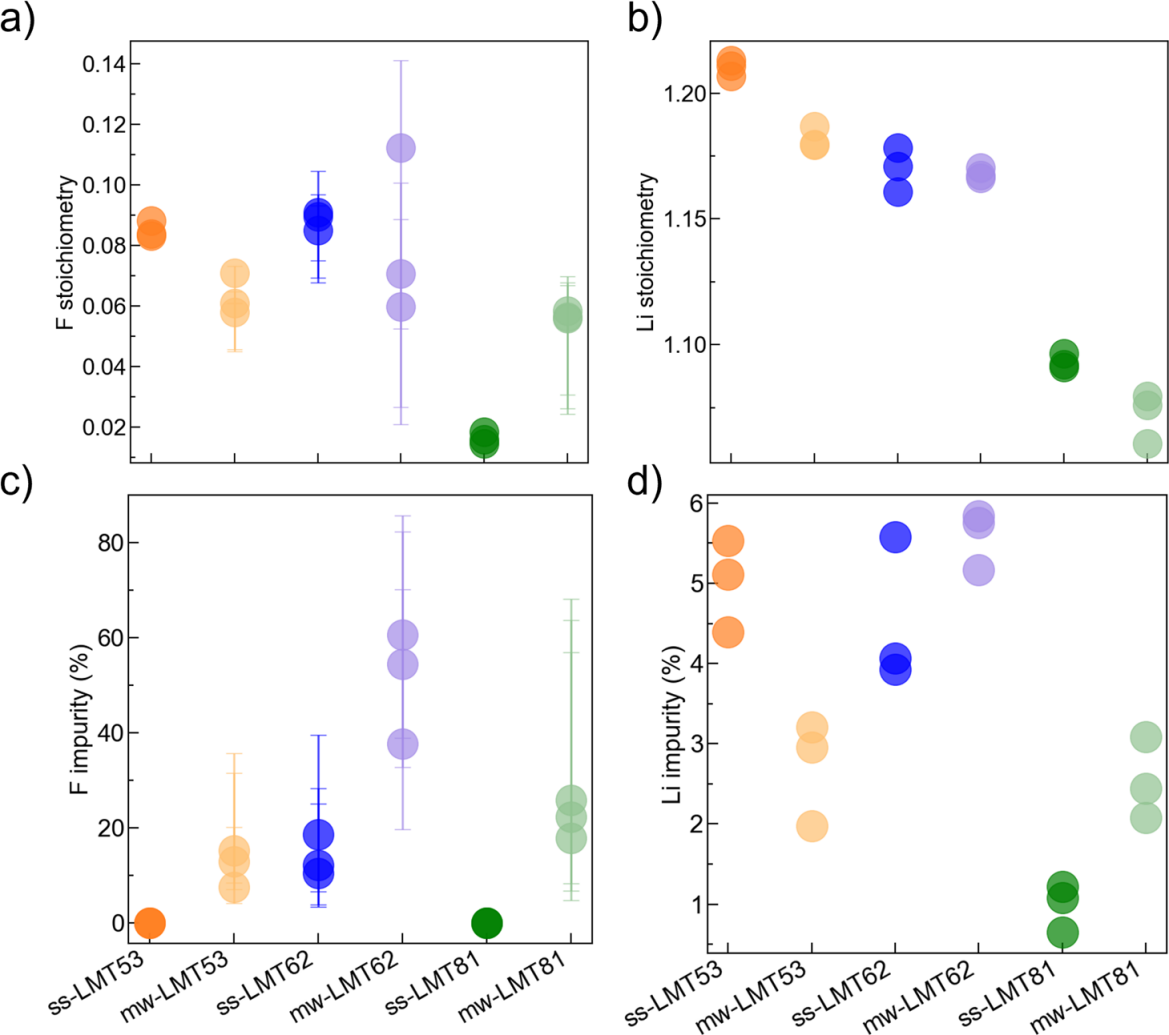
(a,b) F and Li contents in the DRX phase and (c,d) molar fraction of F and Li (in %) present in impurity phases in LMT53, LMT62, and LMT81 samples prepared via solid-state and microwave synthesis after optimization of the synthesis parameters. Triplicates of all compositions of interest were prepared to account for batch-to-batch variation. Error bars are set by the upper and lower bound of fluorination based on the ^19^F ssNMR results and computed as described in Supplementary Note 2.

To account for possible batch-to-batch variation, triplicates were prepared for each of the six samples (ss-LMT53, ss-LMT62, ss-LMT81, mw-LMT53, mw-LMT62, and mw-LMT81). The cation and F composition of the DRX phase in each sample, listed in Table S2, was determined using the hybrid experimental–computational method presented earlier. While this method does not provide an oxygen content, the total anion stoichiometry can be assumed as close to 2 for reasons detailed in our previous work.^33^ For all samples, the Mn to Ti ratio obtained from ICP analysis was found to be very close to the target value. Thus, the compositional analysis focuses hereafter on the Li and F contents in DRX and impurity phases (Figure 3). Overall, the Li and F contents obtained for triplicate samples are in good agreement, where the observed batch-to-batch spread in DRX fluorination is slightly wider for microwave synthesized than for solid-state synthesized samples. However, this is to be expected since the exact reaction temperature cannot be controlled in our microwave setup.

For the LMT53 composition with a target F stoichiometry of 0.1, the actual F content determined for the solid-state synthesized samples ranges from 0.083 to 0.088, while the microwave synthesized samples range from 0.058 to 0.071. These values are relatively close to the target value, especially for the solid-state synthesized samples. The fraction of F in the initial precursor mixture that is not incorporated either remains as LiF impurities or is vaporized (Table S3). Similarly, the Li content for LMT53 synthesized via both solid-state and microwave are close to the target value of 1.2, where a slight decrease in Li excess for mw-LMT53 corresponds well to the reduced fluorination observed. For LMT62, the target F content is increased to 0.2 to allow for a greater amount of redox-active Mn, and actual F contents in the range of 0.085–0.091 for solid-state synthesized samples, and of 0.060–0.112 for microwave synthesized samples, are obtained. Regardless of the synthesis method, less than half of the F in the precursor mixture is incorporated into the LMT62 structure, and a significant drop in Li content below the expected value of 1.2 is observed. Finally, LMT81 has the highest Mn content, a reduced amount of Li excess, and a target F content of 0.1. Solid-state synthesized samples show very minimal fluorination on the order of 0.014–0.018, while microwave synthesized samples show significantly more fluorination on the order of 0.056–0.058.

These results indicate that the amount of F that can be incorporated into Mn^3+^-Ti^4+^ DRX (with a Mn content ≥ 0.5) using a standard solid-state or a microwave synthesis route is below F_0.10_. Hence, assuming a F content close to target for systems designed with a fluorination level *F* > 0.1, e.g., Li_1.2_Mn_0.6_Ti_0.2_O_1.8_F_0.2_ (LMT62), is erroneous. Notably, given a certain synthesis method, no significant difference is observed between the amount of F incorporated into LMT53 and LMT62, despite their different Mn/Ti ratios. The low fraction of F incorporated into the LMT62 DRX phase is charge–balanced by a reduced Li^+^ content, as shown in Figure 3b, along with the possible oxidation of small amounts of Mn^3+^ to Mn^4+^. Regarding solid-state synthesized DRX, the amount of F integrated into the bulk structure depends on composition and generally decreases with increasing Mn content, in good agreement with our recent work,^43^ so much so that ss-LMT81 shows almost no fluorination. Interestingly, microwave synthesized LMT81 exhibits a significant fluorination of around 0.06, which may be attributed to the higher temperatures reached with microwave heating, possibly enabling higher fluorine incorporation due to entropic stabilization. Rapid microwave heating seems to be most advantageous for the fluorination of LMT81, since higher fluorination levels can be reached via standard solid-state synthesis for LMT62 or LMT53. In fact, the actual F contents in mw-LMT53, mw-LMT62, and mw-LMT81 are all similar, suggesting that despite the high temperatures reached during the microwave process, F incorporation is kinetically limited due to the rapid 5 min synthesis. This is also evidenced by the significantly higher amount of LiF impurities present in the microwave samples, as shown in Figure 2c and Figure 3c. The fraction of F present as LiF impurities in mw-LMT53, mw-LMT62, and mw-LMT81 is roughly 30%, 80%, and 60%, while that in ss-LMT53, ss-LMT62, and ss-LMT81 is 0%, 30%, and 0%, respectively. Increasing the microwave reaction time may allow for greater DRX fluorination and reduce LiF impurities, but reactions beyond 5 min result in melting of the pellet with our current setup.

Finally, it is important to note the pivotal role of CEMC simulations in allowing the DRX F content to be determined with accuracy in the presence of a high Mn content or when significant F-containing impurities are present. In particular, for mw-LMT62 and mw-LMT82 samples, a large fraction of DRX F environments becomes NMR invisible, and if a significant fraction of LiF is also present, experimental results alone will provide a wide range of possible F contents (see Table S2), giving rise to the large error bars shown in Figure 3a,c. The compositional analysis presented herein has identified a limit to the incorporation of F into Mn^3+^- and Ti^4+^-based DRX with a Mn content ≥ 0.5 and has enabled the quantification of Li- and F-containing impurities in the samples of interest, paving the way to a better understanding of their impact on the electrochemical performance.

### Relation between DRX Composition and Electrochemical Performance

**Figure 4:**
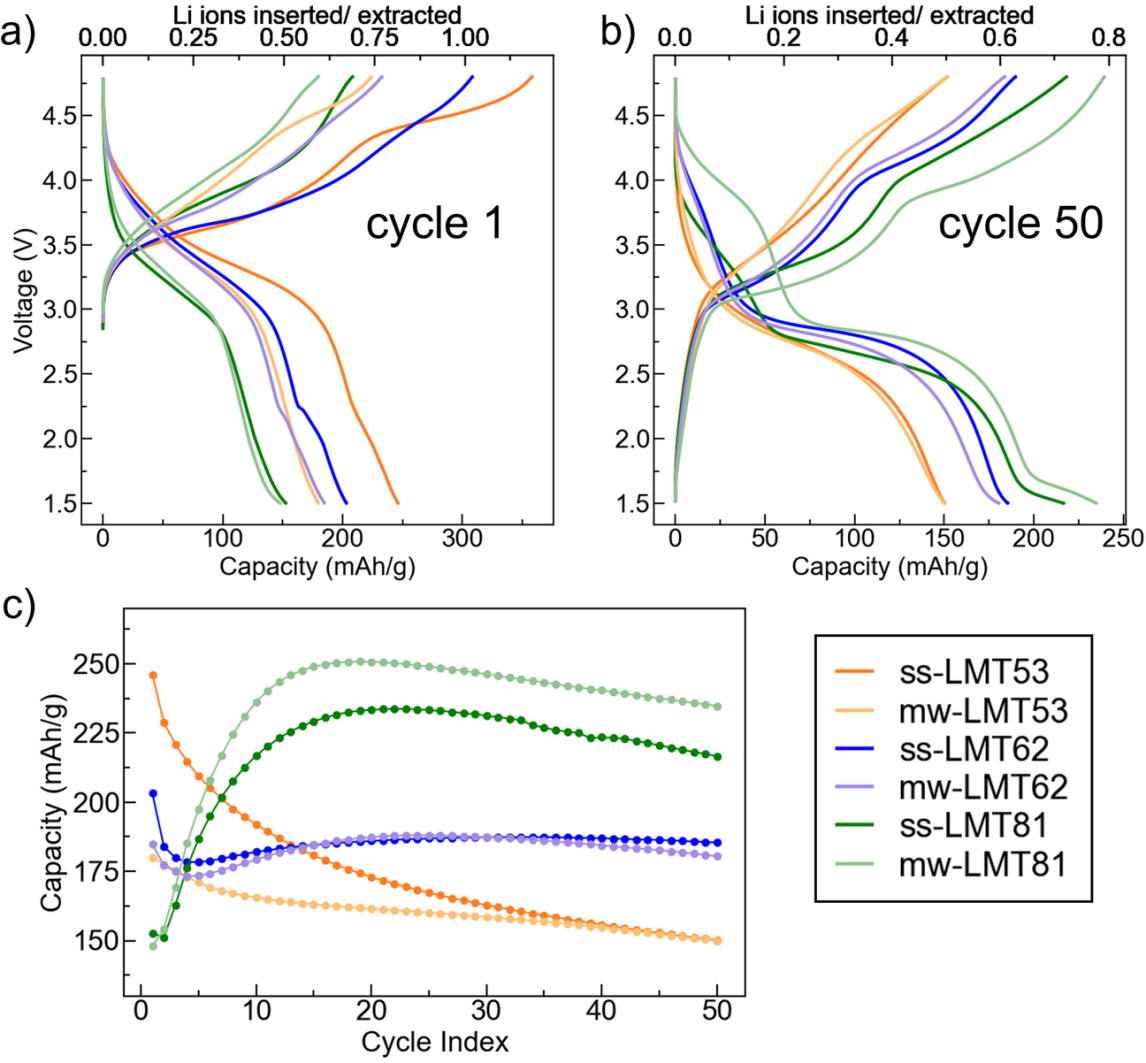
Electrochemical performance of the solid-state and microwave synthesized LMT53, LMT62, and LMT81 cathodes in Li half cells. (a,b) Galvanostatic charge–discharge voltage profiles at cycle 1 and cycle 50 and (c) discharge capacity as a function of cycle number for the three DRX compositions of interest.

The cycling performance of LMT53, LMT62, and LMT81 cathodes prepared by solid-state and microwave syntheses was evaluated in Li half cells. DRX powders were carbon coated and downsized in a ball mill to improve conductivities, and SEM images of postprocessed composite powders show similar particle size distributions (Figure S10). Cells were cycled galvanostatically at 25 °C, over a 1.5–4.8 V potential window, and at a 20 mA/g rate for 50 cycles. The galvanostatic curves obtained during the first and 50th cycle are shown in Figure 4a and b, respectively, while Figure 4c shows the specific capacity against cycle number for all samples of interest. Additional galvanostatic profiles, differential capacity curves, and plots showing the evolution of the operating voltage, energy density, voltage hysteresis, and Coulombic efficiency with cycle number are shown in Figures S11–S13.

Out of the three DRX compositions considered here, the LMT53 cathode exhibits the poorest capacity retention; however, its initial capacity and capacity retention are found to depend on the exact composition and synthesis method. The ss-LMT53 cathode exhibits a high initial capacity of 246 mAh/g, which decreases to 150 mAh/g after 50 cycles, corresponding to a capacity retention of 60%. The mw-LMT53 cathode, on the other hand, exhibits a much lower initial capacity of 180 mAh/g and a less pronounced capacity drop with 150 mAh/g observed after 50 cycles, corresponding to a capacity retention of 83%. Notably, the Mn-based redox capacity of LMT53 amounts to 160 mAh/g, suggesting that a significant fraction (85 mAh/g) of the initial capacity of the solid-state synthesized sample must rely on anion-based redox, which has been associated with lattice oxygen loss and surface densification^31,44^ and likely explains its rapid drop in performance. The evolution of the anion redox contribution to the ss-LMT53 capacity is clear from Figure S12, where the initial d*Q*/d*V* peak at 4.5 V on charge, attributed to anion redox activity, rapidly decreases in intensity and disappears completely by cycle 50. In contrast, the high voltage feature in the d*Q*/d*V* plot for mw-LMT53 disappears after the first few cycles, presumably due to initial cathode–electrolyte interphase formation and stabilization, and slowly grows back again upon extended cycling, suggesting an increased contribution from bulk anion redox processes. These contrasting electrochemical behaviors may stem from slight compositional differences between ss-LMT53 and mw-LMT53. The higher initial capacity of ss-LMT53, compared to mw-LMT53, may partly be accounted for by its higher Li excess content, resulting in a more extensive network of Li-diffusing (0-TM) channels that could lead to a greater fraction of extractable Li.^45^

For LMT62 and LMT81, a partial structural transformation occurs during cycling. At the local scale, the initial disordered arrangement of cations in the rocksalt lattice partially evolves into a spinel-like cation ordering, referred to as the “δ phase” in previous reports on related Mn-rich DRX compounds (e.g., with a Mn content ≥ 0.6).^25,26,46^ Notably, the δ phase transformation has been found to facilitate Li transport within and extraction from the cathode particles. Indeed, clear capacity activation is observed for LMT62 and LMT81 over the first few cycles and concurrently with δ phase formation, as shown in Figure 4. In comparison to LMT53, the electrochemical performance of LMT62 does not depend significantly on the synthesis route. For LMT62, besides differences in initial capacities (204 mAh/g for ss-LMT62 and 185 mAh/g for mw-LMT62), cathodes prepared via solid-state and microwave synthesis behave very similarly, with a capacity of 187–188 mAh/g after around 25–30 cycles, which fades to 185 or 180 mAh/g by cycle 50, as shown in Figure 4c. These similarities are reflected in the d*Q*/d*V* plots shown in Figure S12, where the solid-state and microwave samples undergo a steady decline in the discharge voltage. The evolution of the capacity of LMT62 with cycling reflects both the beneficial bulk structural transformation that leads to a general increase in capacity and surface structural degradation mechanisms exacerbated by the high (4.8 V) and low (1.5 V) voltage cutoffs used here. Past an initial capacity drop during the first few cycles, the slow transformation to the δ phase results in an increase in capacity over ∼30 cycles. After that, degradation effects begin to dominate, and the capacity slowly fades. For LMT81, the higher Mn content induces a more rapid and extensive transformation to the δ phase with cycling, consistent with a prior report.^46^ ss-LMT81 exhibits an initial capacity of 153 mAh/g, which reaches a maximum of 233 mAh/g after around 20 cycles and fades to 217 mAh/g at cycle 50. The capacity of mw-LMT81 initially increases from 148 to 251 mAh/g after 20 cycles and later fades to 235 mAh/g after 50 cycles. Past peak capacity activation (20 cycles), the mw-LMT81 cathode exhibits an additional ∼20 mAh/g of capacity compared with its solid-state synthesized counterpart, which may result from the higher fluorine content in mw-LMT81. We note that although ss-LMT81 has a slightly higher Li content, due to the in situ δ phase transformation for high-Mn content DRX, Li excess becomes significantly less impactful in terms of improving Li percolation networks. Comparing the voltage profiles in Figure 4a,b, much of the additional capacity obtained from mw-LMT81 comes from its longer low-voltage tail. The differential capacity analysis (Figure S12) indicates that the low voltage cathodic peak is shifted to higher potentials in the microwaved version of LMT81, allowing for greater capacity to be extracted within the 1.5–4.8 V potential range. Aside from their different low voltage behaviors, ss- and mw-LMT81 also differ at the pseudoplateau regions at ∼3 and ∼4 V. After 50 cycles, the pseudoplateaus are more pronounced for mw-LMT81, as evidenced by the sharper d*Q*/d*V* peaks in Figure S12, suggesting a more extensive growth of the spinel-like δ phase in this compound. While the ∼4 V pseudoplateau initially grows over the first 20 cycles and subsequently fades for ss-LMT81, it steadily grows upon extended cycling for mw-LMT81. As a result, mw-LMT81 shows remarkably minimal voltage fade past 20 cycles, unlike ss-LMT81, which exhibits a steady decrease in the average operating potential (Figure S13a). In summary, for high Mn DRX cathodes, the extent to which the disordered structure transforms to the δ phase during cycling is a determinant of long-term electrochemical performance. While the Mn content in the cathode is the single most important factor toward the formation of a δ phase, the differences in electrochemical performance observed between ss- and mw-LMT81 suggest that other factors, such as Li excess, F content, and short-range order, may also contribute to the overall structural evolution of the cathode.

Regardless of the synthesis method, the medium- and long-term electrochemical performance improves as the Mn content increases from LMT53 to LMT62 and to LMT81. Specifically, the 50 cycle capacity increases from ∼150 mAh/g to ∼180 mAh/g and to ∼220–235 mAh/g across the series of compounds. This improvement largely results from the electrochemically induced phase transformation and associated capacity activation as well as reduced reliance on anion-based redox processes in LMT62 and LMT81. Notably, the amount of F in the DRX phase appears to be a secondary predictor of the long-term performance, as evidenced by the fact that the three microwave synthesized samples contain very similar F contents yet exhibit very different electrochemical behaviors. Nevertheless, the superior electrochemical performance of mw-LMT81 compared with ss-LMT81 may be related to its higher F content.

Finally, to investigate the impact of impurity phases present in the as-prepared cathode sample on the electrochemical performance, we compare ss- and mw-LMT62 with almost identical DRX stoichiometries but significantly different amounts of the F-containing impurity phase (i.e., LiF). LiF impurities make up ∼50% and ∼20% of the entire F molar content in mw- and ss-LMT62 samples, respectively, corresponding to weight fractions of 1.2% and 0.5%, respectively. Despite a more than 2-fold increase in the amount of F impurities in the microwave-synthesized DRX sample, no significant difference in cell performance (Figure 4) is observed. For LMT81, ∼20% of the F is present as LiF in the mw-LMT81 sample (corresponding to a LiF weight percent of 0.4%), whereas no LiF is present in ss-LMT81, but the microwave synthesized cathode performs significantly better. Overall, provided that the amount of LiF in the cathode sample is sufficiently low (all syntheses were carefully optimized in this work), this impurity phase does not seem to significantly impede the electrochemical performance.

In this study, a hybrid experimental-computational method was developed to determine the stoichiometries of DRX cathodes. This method was used to optimize solid-state and microwave synthesis parameters for three Mn^3+^- and Ti^4+^-based DRX, namely, Li_1.2_Mn_0.5_Ti_0.3_O_1.9_F_0.1_ (LMT53), Li_1.2_Mn_0.6_Ti_0.2_O_1.8_F_0.2_ (LMT62), and Li_1.1_Mn_0.8_Ti_0.1_O_1.9_F_0.1_ (LMT81), to maximize their phase purity and the amount of F integrated into their bulk structures. Our results indicated that, for this family of compounds, the F content is always below F_0.10_. Interestingly, we found that microwave synthesis was more effective at fluorinating Mn-rich DRX compounds and results in a F content of F_0.06_ for LMT81, in contrast to solid-state synthesis which led to almost no fluorination (< F_0.02_). The synthesis conditions and resulting compositions of the DRX cathodes of interest were carefully related to their electrochemical performance. For LMT53, LMT62, and LMT81, a high Mn content was found to be the single most important factor for high-capacity and long-term stability, even if it resulted in limited fluorination or a lower Li excess content. Further, at a fixed F content, a higher Mn content was found to increase capacity and improve its retention during long-term cycling, in part due to a partial phase transformation from the initial DRX phase to a δ phase with a local spinel-like cation ordering. A small amount of F was found to be beneficial for capacity and capacity retention, even at high Mn contents, as indicated by the ∼20 mAh/g additional capacity observed for mw-LMT81 as compared with ss-LMT81 after initial capacity activation. Other factors, such as Li excess and/or the initial short-range order in the DRX phase, likely also impact the structural transformation and therefore the electrochemical behavior of Mn-rich DRX cathodes.

## Supporting Information Available

A detailed description of the hybrid computation–experimental method to determine DRX stoichiometries; optimization of solid-state and microwave DRX synthesis with supporting XRD and stoichiometry plots; fits of ssNMR spectra; table of final DRX stoichiometries; more detailed electrochemical cycling data (PDF)
