# Supplementary material for: The Limited Incorporation and Role of Fluorine in Mn-rich Disordered Rocksalt Cathodes
Source: ACS Energy Lett. 2024 May 30;9(6):3027–35. doi: 10.1021/acsenergylett.4c01075 (PMC11190981; doi:10.1021/acsenergylett.4c01075)
Supplement: Supplementary file 1 — nz4c01075_si_001.pdf [file nz4c01075_si_001.pdf]

Supporting Information: The Limited Incorporation and Role of Fluorine in Mn-rich Disordered Rocksalt Cathodes

Vincent C. Wu,[†] Peichen Zhong,[‡,¶] Julia Ong,[†] Eric Yoshida,[†] Andrew Kwon,[†] Gerbrand Ceder,[‡,¶] and Raphaële J. Clément*[*,†]

†*Materials Department and Materials Research Laboratory, University of California, Santa Barbara, Santa Barbara CA 93106, USA*

‡*Department of Materials Science and Engineering, University of California Berkeley, Berkeley CA 94720, USA*

¶*Materials Sciences Division, Lawrence Berkeley National Laboratory, Berkeley CA 94720, USA*

E-mail: rclement@ucsb.edu

Methods

Computational Methods

Density functional theory calculations

All the DFT calculations were performed using the Vienna ab initio simulation package (VASP) using the projector-augmented wave method,[1] a plane-wave basis set with an energy cutoff equal to 680 eV, and a reciprocal space discretization of 25 k-points per $Å^{-1}$. All the calculations were converged to 10^{-6}eV in total energy for electronic loops and 0.02 eV/$Å$ in interatomic forces for ionic loops. We relied on the regularized strongly constrained and appropriately normed meta-GGA exchange-correlation functional (r^2SCAN),[2] which is believed to better capture cation-anion hybridization and Li-coordination. r^2SCAN has better computational efficiency performance than the earlier version of SCAN.[3]

Monte Carlo cluster expansions

The cluster-expansion (CE) technique was used to study the configurational thermodynamics of materials in which sites can be occupied by multiple cations and has been applied to study the Li-vacancy configuration in layered materials[4] and the cation SRO in DRX compounds.[5–7] The CE expands the energy of multicomponent disordered rocksalt materials as a sum of many-body configurational interactions:

$$E(\sigma) = J_0 + \sum_i J_i\sigma_i + \sum_{i,j} J_{ij}\sigma_i\sigma_j + \sum_{i,j,k} J_{ijk}\sigma_i\sigma_j\sigma_k + ..., \tag{1}$$

where σ_i is the occupancy of different species (indicator basis site function) and J refers to the effective cluster interaction (ECI) using the sinusoid basis.[4] For the simulation of atomic orderings, a cluster-expansion Hamiltonian was generated in the chemical space of Li^+-Mn^{3+}-Mn^{4+}-Ti^{4+}- O^{2-}-F^-, with pair interactions up to 7.1 Å, triplet interactions up to 4.0 Å, and quadruplet interactions up to 4.0 Å based on a primitive cell of the rocksalt

structure with lattice parameter $a = 3$Å. In total, 162 ECIs (including the constant term J_0) were defined, and the CE Hamiltonian was fitted with 563 different structures. As the CE Hamiltonian was defined on a high-dimensional multicomponent system, the ECIs were fitted using the appropriate method to address the complexity-induced over-fitting.[8,9] The ECIs were determined with the optimal sparseness and cross-validation error (< 8 meV/atom) with a $\ell_0\ell_2$-norm regularized regression.[10] We refer readers to Ref.[11] for details of CE in ionic systems. To simulate atomic orderings at equilibrium, we used canonical Monte Carlo simulation with the Metropolis–Hastings algorithm. Overall, 1,000 representative structures (640 atoms per structure) were sampled from the equilibrium ensemble. We used `smol` for the CEMC simulations[12] and `pymatgen` for the structure processing.[13]

Materials synthesis

Solid-state synthesis of DRX

A standard solid-state synthesis protocol was used to synthesize LMT53, LMT62, and LMT81. Stoichiometric amounts of Li_2CO_3, LiF, Mn_2O_3, and TiO_2 precursors were used, with 10% Li excess to compensate for possible Li loss. Precursor powders were mixed via wet ball-milling with ethanol in a planetary ball-mill at 300 rpm for 6 hours. The resulting slurry was dried to form a powder, and pressed into 200 mg pellets. The pellets were then heated at targeted temperatures for specified reaction times under Ar gas flow in a tube furnace, after which the furnace hood was opened and pellets were allowed to cool naturally. Optimized synthesis temperatures for LMT44, LMT53, and LMT62 were 800°C, 900°C, and 1000°C, respectively; optimized calcination times were 12 h for all compositions.

Microwave synthesis of DRX

For microwave synthesis of LMT53, LMT62, and LMT81, stoichiometric amounts of Li_2CO_3, LiF, Mn_2O_3, and TiO_2 precursors were used; no Li excess was added. Precursors were mixed and pelletized according to the same procedure used for solid-state synthesis, where precursor

powders were ball-milled with ethanol at 300 rpm for 6 hours, dried, and then pressed into 200 mg pellets. A double crucible setup was used for microwave synthesis, where a small alumina crucible was placed inside a larger crucible filled with 5 g of activated charcoal. The precursor pellet was placed in the small alumina crucible on top of a layer of sacrificial precursor powder to prevent a reaction between the alumina surface and the pellet. The entire setup was placed in a conventional 1200W microwave, and heated at appropriate times and microwave power in an ambient air atmosphere. Upon termination of microwaves, the pellet was immediately quenched into a beaker of distilled water to stabilize the DRX phase. The DRX pellet was then dried on a hot plate and ground into powder. Optimized microwave power values for LMT44, LMT53, and LMT62 were 600W, 480W, and 480W, respectively, and optimal synthesis times were 5 min for all compositions.

Characterization

Powder X-ray diffraction data for all samples were collected using a laboratory-source Pananlytical Empyrean diffractometer with Cu Kα radiation in reflection geometry. The TOPAS software suite was used for Rietveld refinements of data sets.[14] Bulk chemical compositions were determined via ICP (Agilent 5800 ICP-OES) and fluoride selective ion electrode (Cole-Parmer) measurements. DRX samples were digested in a mixture of nitric acid and hydrochloric acid. For ICP, the digested solutions were diluted with distilled water. For F-ISE measurements, the solutions were diluted using a sodium acetate buffer and a fluoride ionic strength adjuster solution (TISAB, Cole-Parmer).

Solid-state nuclear magnetic resonance spectroscopy

Solid state ^{7}Li and ^{19}F NMR spectra were acquired at B_0 = 2.35 T (100 MHz for ^{1}H) using a wide bore Bruker BioSpin spectrometer equipped with a DMX 500 MHz console and a custom-made 1.3 mm single X-broadband magic angle spinning (MAS) probe tuned to ^{7}Li (38.9 MHz) or ^{19}F (94.1 MHz). Samples were loaded into 1.3 mm zirconia rotors and closed

using Vespel caps. The samples were spun at the magic angle (MAS) at ν_R= 60 kHz using dry nitrogen. ^{7}Li and ^{19}F chemical shifts were externally referenced against 1M aqueous LiCl (^{7}Li $\delta_{iso} = 0$ ppm) and NaF (^{19}F $\delta_{iso} = -118.14$ ppm) solutions, respectively. Both ^{7}Li and ^{19}F MAS NMR spectra were obtained using a rotor synchronized spin-echo sequence (90° - τ_R - 180° - τ_R) with 90° radio frequency (RF) pulses of 0.45 μs and 0.30 μs, respectively. For ^{7}Li quantitative spin-echo spectra ($\tau_R = 1$ rotor period), a total of 64 transients were averaged with a recycle delay of 20 sec which was enough to reach full relaxation of ^{7}Li signals. Fully relaxed ^{19}F NMR spectra were obtained with a recycle delay of 20 s and signals were averaged over 256 transients with a presaturation step resulting in an acquisition time of 1 h 25 min per sample. To enhance the signal-to-noise ratio of ^{19}F paramagnetic signals, a much shorter recycle delay of 50 ms was used which allowed a larger number of transients (32,768) to be acquired over a reasonable amount of time (30 min per sample). Solid-state NMR data were processed using Bruker TopSpin 3.6.0 and spectra were fit using an in-house developed python script.

Electrochemistry

Electrochemical performance of DRX was tested in coin cells assembled in an Ar filled glovebox. As-synthesized DRX powders were first carbon coated and downsized by ball milling with Super C65 at 400 rpm for 6h in a planetary ball mill. DRX cathode films were then produced by mixing the post-processed, carbon coated DRX powders with polytetrafluoroethylene (PTFE) such that the active material: carbon: binder ratio was 70:20:10. Films were then rolled out and punched to form 6.35 mm diameter discs with loading densities of 5-6 mg/cm^2. CR2023-type coin cells were assembled using the cathode film and a Li metal anode. 1M $LiPF_6$ in ethylene carbonate and dimethyl carbonate (EC/DMC with a 1:1 volume ratio) electrolyte was used, along with a glass fiber (Whatman GF/D) separator. Coin cells were cycled in a temperature controlled chamber (Neware MHW-25-S) set to 25°C using a Biologic VMP-3 tester.

Supplementary Note 1:

Hybrid experimental-computational methodology to determine DRX stoichiometry

Does fluorination impact DRX? The role of fluorine in DRX cathode A flowchart summarizing the methodology used to determine DRX stoichiometries is shown in Figure S1. The method described below builds upon and extends the experimental approach recently proposed by some of us[15] to determine the composition of DRX samples, and improves the accuracy of F content predictions.

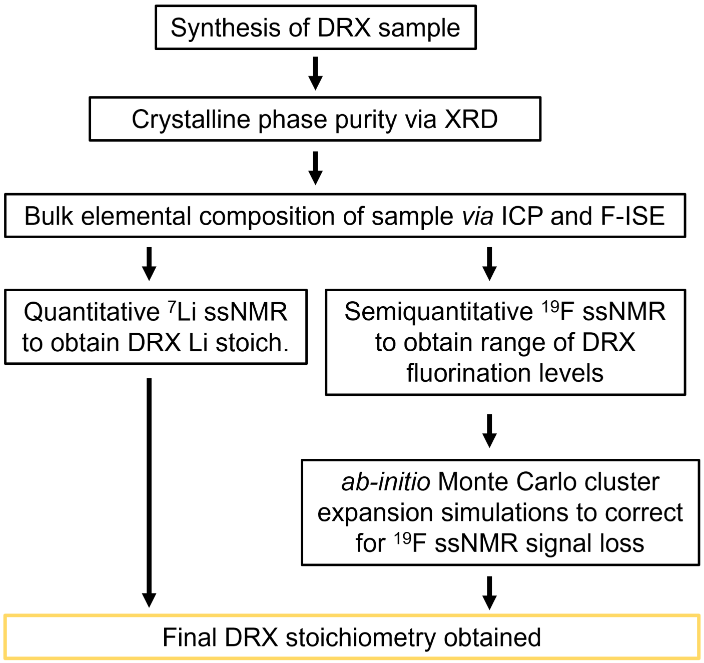

Figure S1: Flowchart of process to determine DRX stoichiometry.

In the first step, the as-synthesized sample is examined using powder XRD to identify potential crystalline impurity phases. Samples that contain transition metal oxide or oxyfluoride phases other than the disordered rocksalt phase of interest are discarded, as are samples containing a significant amount of crystalline impurities, such as LiF, Li_2CO_3, or Li_2O.

For samples that are (close to) phase pure by XRD, the composition of the sample is analyzed in a second step using ICP and a F-ISE, which provide the overall ratio of cationic species and the F content, respectively. Combining XRD, ICP, and F-ISE results, it is clear that all transition metal species are integrated into the DRX structure, but the presence of (at least partially) amorphous Li- and F-containing impurity phases means that such phases must be quantified in order to obtain the Li and F contents in the DRX phase.

Li-containing impurity phases (crystalline and/or amorphous) can be quantified using ^{7}Li ssNMR. A typical ^{7}Li spectrum obtained on a DRX sample is shown in Figure S2, where two types of resonances are observed: a sharp resonance centered around 0 ppm corresponding to Li in diamagnetic environments (red dotted line), and broader resonances associated with paramagnetic Li environments (blue dotted lines). The diamagnetic signal either results from diamagnetic impurity phases in the sample, such as Li_2CO_3 and Li_2O,[16] or from diamagnetic domains in the DRX phase.[15] However, for the compositions of interest to this work, the probability of forming diamagnetic Li environments in the DRX structure is negligible due to the high concentration of paramagnetic metal species (here, redox-active Mn), and the diamagnetic signal can therefore be assigned to Li-containing impurity phases. The broad paramagnetic signals can be attributed to Li species in the DRX phase, which are in close proximity to the redox-active metals (referred to as M hereafter), resulting in strong paramagnetic couplings between the ^{7}Li nuclear spin and unpaired electron spins originating from nearby M d orbitals. Those paramagnetic interactions manifest as a large Fermi contact shift and significant broadening of the resonance. Additionally, the extensive disorder of cationic and anionic species in DRX compounds leads to a large number of possible Li local environments, each with a (slightly) different shift, and extensive overlap of the resulting broad paramagnetic resonances. By fitting the sharp diamagnetic signal and broad paramagnetic resonances (Figure S2), the molar fraction of Li in impurity phases can be determined and used to scale ICP results and derive the Li stoichiometry of the DRX phase.

Similarly to ^{7}Li ssNMR, typical ^{19}F ssNMR spectra obtained on DRX cathode samples consist of broad paramagnetic and sharp diamagnetic resonances. In the case of ^{19}F ssNMR, the sharp diamagnetic peak at -204 ppm is attributed to LiF,[17] and the broader features correspond to F species in paramagnetic (DRX) environments. Unlike Li, F can be directly bonded to paramagnetic M species which, compounded with the high gyromagnetic ratio (γ) of ^{19}F, results in extremely strong paramagnetic interactions and extremely fast ^{19}F ssNMR signal decay. Thus, F species bonded to at least one paramagnetic Mn ion are effectively invisible by NMR, and integration of the ^{19}F ssNMR signals underestimates the amount of F in DRX environments. In order to quantify the amount of F in the DRX structure from ^{19}F ssNMR, the fraction of "NMR (in)visible" F environments must be determined. Here, *ab initio* cluster expansion Monte Carlo (CEMC) simulations allow us to predict the equilibrium distribution of F environments in the DRX compounds of interest at typical synthesis temperatures. From this, the integrated paramagnetic ^{19}F ssNMR signal intensity is scaled to account for signal loss, and the DRX F content is obtained by combining the F-ISE and ^{19}F ssNMR results, as discussed in Supplementary Note 2.

Supplementary Note 2: Analysis of the ^{19}F ssNMR results to quantify the F content in the DRX phase

As mentioned in Supplementary Note 1, while the integration of ^{19}F ssNMR signals should, in theory, provide quantitative insight into the distribution of F species in the DRX phase and in impurity phases, F species directly bonded to Mn cannot readily be observed, resulting in an underestimation of DRX fluorination. Two possible approaches to scale the integrated intensity of the paramagnetic ^{19}F ssNMR signal (p) are presented below, which either rely on simple probability calculations, or on CEMC simulations of the distribution of F environments in the structure.

The experimentally determined fraction of F in the DRX, F_{exp}, is calculated by dividing the integrated paramagnetic signal intensity p by the total signal intensity, which is the sum of p and the integrated diamagnetic signal intensity d (see Figure S2).

$$F_{exp} = \frac{p}{p+d} \tag{2}$$

Given that p is underestimated experimentally, F_{exp} provides a lower bound for the amount of F in the DRX structure. An adjusted (quantitative) paramagnetic intensity, P, and a scaled DRX F fraction, F_{scaled}, can be determined if the fraction of NMR-visible F environments in the DRX phase is known. The fraction of NMR-visible F sites is hereafter referred to as *F-Mn(0)*, as none of the six nearest-neighbor cations (C) surrounding those F species can be Mn. The adjusted paramagnetic intensity P is calculated using the equation $P = p/F\text{-}Mn(0)$. Subsequently, F_{scaled} can be obtained using:

$$F_{scaled} = \frac{P}{P+d} = \frac{\frac{p}{P(all\, C \neq Mn)}}{\frac{p}{P(all\, C \neq Mn)} + d} \tag{3}$$

Here, we use CEMC simulations of a 640-atom supercell to model the distribution of environments (and any short-range ordering) in LMT53, LMT62, and LMT81. Direct counting

of the F environments provides *F-Mn(0)* at the simulation temperature, from which the fraction of F in the DRX can be obtained by scaling the integrated paramagnetic signal intensity. The DRX fluorination level is then calculated by scaling the total F content in the DRX sample (obtained from F-ISE measurements) by F_{scaled}.

If CEMC simulations are not available, an upper bound for the amount of F in the DRX structure can be approximated by computing *F-Mn(0)* assuming a random distribution of species within the DRX structure. The assumption of complete disorder leads to an overestimation of the number of Mn-F bonds, and thereby of the fraction of NMR invisible F environments, and to an upper bound for the DRX F content.[15]

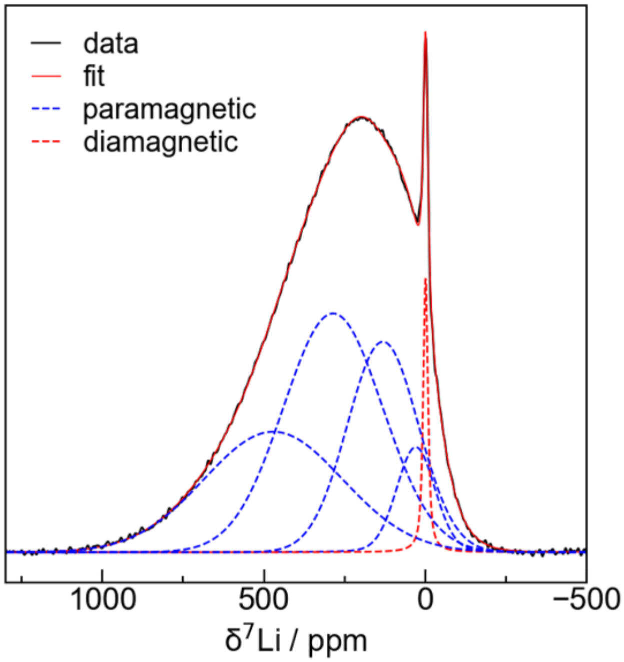

Figure S2: Representative fit of the ^{7}Li ssNMR spectrum collected on ss-LMT62 with a short 50ms recycle delay, illustrating the broad paramagnetic and sharp diamagnetic signals corresponding to Li in the DRX and in Li-containing impurity phases, respectively.

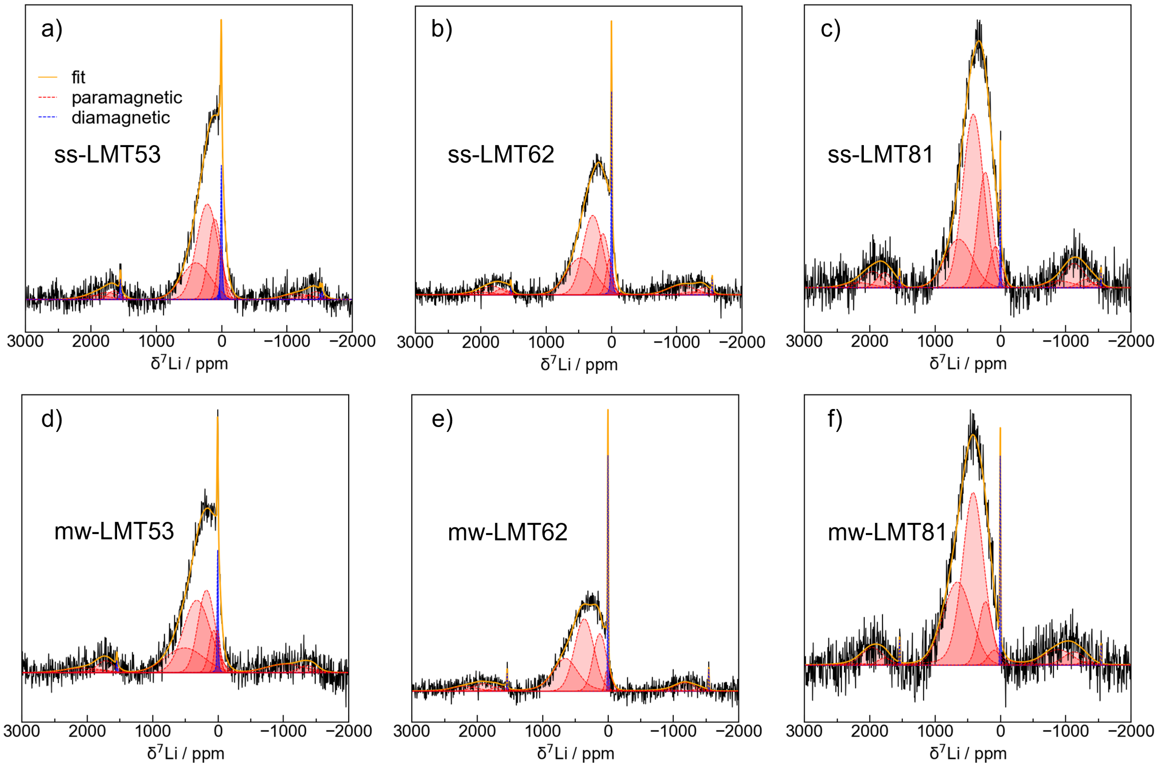

Figure S3: Fits of ^{7}Li ssNMR spectra for optimized solid-state and microwave synthesized LMT53, LMT62, and LMT81 samples. ssNMR spectra were acquired at 2.35 T with a magic angle spinning (MAS) speed of 60 kHz with a long 20 sec recycle delay to ensure sufficient relaxation of diamagnetic environments. Fits were performed using an in-house developed python program. For each DRX sample, an initial fit was first done on a ssNMR spectrum acquired with a short recycle delay of 50ms to obtain high quality fits to the paramagnetic lineshape (see Figure S2). The model obtained from this fit then used for the final fitting on sufficiently relaxed spectra with a 20 sec recycle delay (shown in this figure), where only component peak widths and magnitudes were allowed to vary. Diamagnetic impurity percentages were extracted by integrating paramagnetic and diamagnetic components of the fit ssNMR spectra, and results for each sample are listed in Table S2.

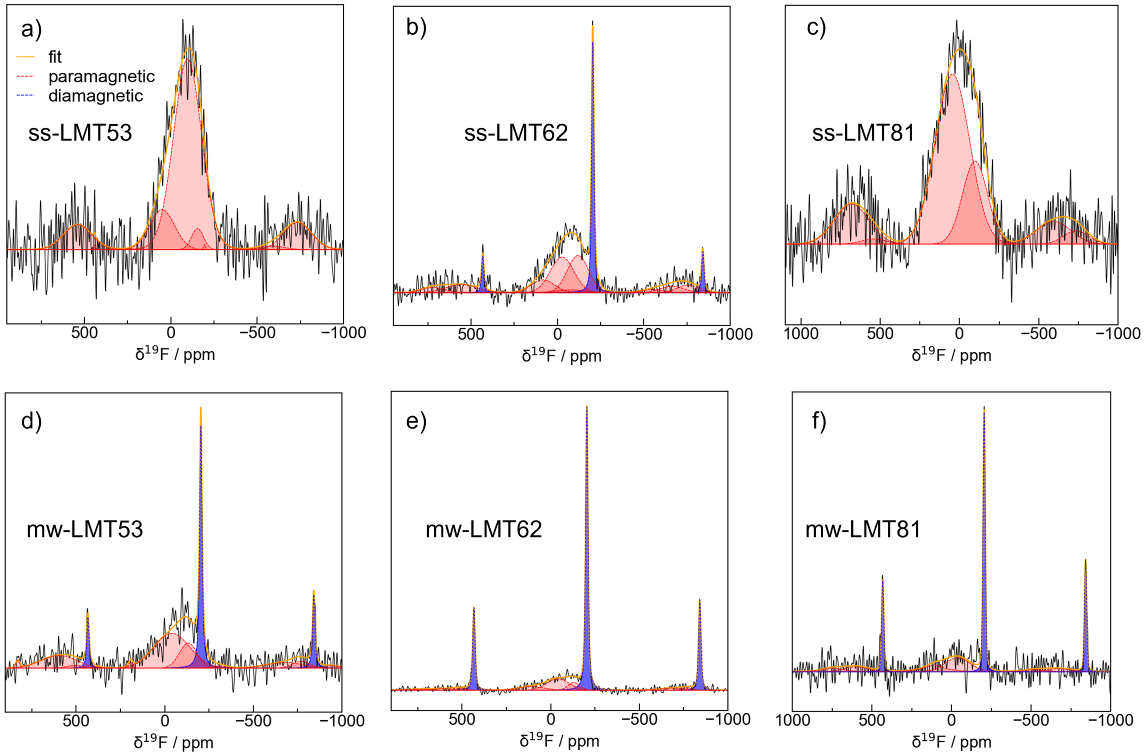

Figure S4: Fits of ^{19}F ssNMR spectra for optimized solid-state and microwave synthesized LMT53, LMT62, and LMT81 samples. ssNMR spectra were acquired at 2.35 T with a magic angle spinning (MAS) speed of 60 kHz. All spectra shown except for ss-LMT81 were acquired with a long 20 sec recycle delay to ensure sufficient relaxation of diamagnetic environments. For ss-LMT81, the spectrum shown was acquired with a 50 ms recycle delay, as no ^{19}F ssNMR signal was observed when a 20 cycle recycle delay was used, due to the almost negligible fluorine content in the sample. Semiquantitative fluorine impurity percentages obtained from these fits are listed in Table S3.

Supplementary Note 3: DRX compositions and temperatures considered for *ab initio* CEMC simulations

As discussed in Supplementary Note 2, in the absence of *ab initio* CEMC simulations, an upper and a lower bound for the amount of F in the DRX can still be obtained from ICP, F-ISE, and ssNMR results assuming a random distribution of species in the rocksalt structure. These bounds were used here to guide input DRX stoichiometries for *ab initio* CEMC simulations, listed in Table S1. We note that, while restrictions on the size of the input supercells and imposed by charge neutrality mean that the supercell composition never truly matches the experimental one, we find that small changes in DRX stoichiometry (including of the F content) do not significantly affect the distribution of F environments. For the microwave synthesized samples, since synthesis was carried out under air, a small amount of Mn^{3+} to Mn^{4+} oxidation may be expected. We therefore carried out a second series of *ab initio* CEMC simulations on DRX compositions mimicking LMT53, LMT62, and LMT81 with 5% Mn^{4+}. We find that a small amount of Mn^{4+} has a minimal effect on short-range order (Figure S5a), hence those Mn^{4+}-containing calculations are not considered further for the analysis of the experimental results. While the *ab initio* CEMC simulations were carried over a wide range of temperatures from 800°C to 1800°C (all simulation results are plotted in Figure S5), the input parameters for the *ab initio* CEMC simulations used to determine the distribution of F environments in the LMT53, LMT62, and LMT81 samples of interest are listed in Table S1.

Table S1: Chemical formulae of the DRX supercells and temperature inputs for the *ab initio* CEMC simulations used to determine the distribution of F environments and F content in the LMT53, LMT62, and LMT81 samples of interest. For the solid-state synthesized samples, the Mn oxidation state was assumed to be 3+, while for the microwave synthesized samples, a mixture of 3+ and 4+ Mn oxidation states was assumed. For solid-state synthesized samples, CEMC simulation temperatures were chosen to be as close as possible to the experimental sintering temperatures. For microwave synthesized samples, a 1600°C CEMC simulation temperature was chosen throughout since the temperature of the DRX pellets was found to be 1200°C a few seconds after microwave heating, suggesting a higher heating temperature closer to 1500-1600°C.

Sample	Reaction conditions	CEMC formula	CEMC temp. (°C)
ss-LMT53 (optimized)	800 °C, 12 h	$Li_{192}{Mn^{3+}}_{80}Ti_{48}O_{304}F_{16}$	800
ss-LMT62 (optimized)	900 °C, 12 h	$Li_{185}{Mn^{3+}}_{101}Ti_{34}O_{304}F_{16}$	800
ss-LMT81 (optimized)	1000 °C, 12 h	$Li_{172}{Mn^{3+}}_{132}Ti_{16}O_{312}F_{8}$	1000
mw-LMT53 (optimized)	720W, 5 min	$Li_{193}{Mn^{3+}}_{77}{Mn^{4+}}_{3}Ti_{47}O_{304}F_{16}$	1600
mw-LMT62 (optimized)	600W, 5 min	$Li_{187}{Mn^{3+}}_{95}{Mn^{4+}}_{4}Ti_{34}O_{304}F_{16}$	1600
mw-LMT81 (optimized)	600W, 5 min	$Li_{175}{Mn^{3+}}_{123}{Mn^{4+}}_{6}Ti_{16}O_{312}F_{8}$	1600
ss-LMT53	800 °C, 2 h	$Li_{192}{Mn^{3+}}_{80}Ti_{48}O_{304}F_{16}$	800
ss-LMT62	900 °C, 2 h	$Li_{185}{Mn^{3+}}_{101}Ti_{34}O_{304}F_{16}$	800
ss-LMT81	1000 °C, 2 h	$Li_{172}{Mn^{3+}}_{132}Ti_{16}O_{312}F_{8}$	1000
mw-LMT53	1200W, 5 min	$Li_{193}{Mn^{3+}}_{77}{Mn^{4+}}_{3}Ti_{47}O_{304}F_{16}$	1600
mw-LMT62	1200W, 5 min	$Li_{187}{Mn^{3+}}_{95}{Mn^{4+}}_{4}Ti_{34}O_{304}F_{16}$	1600
mw-LMT81	1200W, 5 min	$Li_{175}{Mn^{3+}}_{123}{Mn^{4+}}_{6}Ti_{16}O_{312}F_{8}$	1600

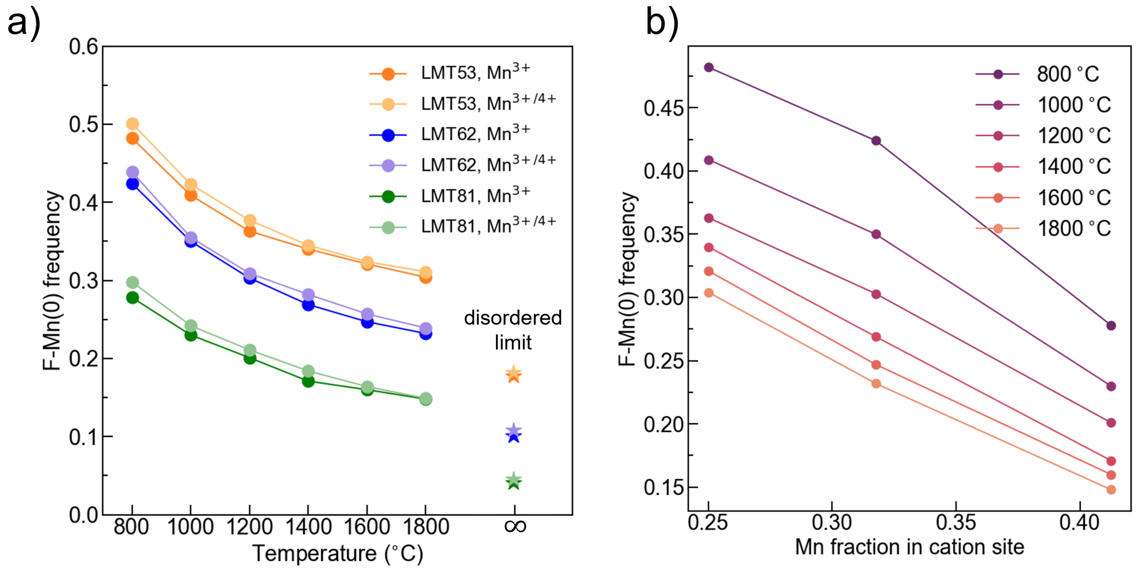

Figure S5: a) Frequency of F species with no nearest-neighbor Mn, *F-Mn(0)*, obtained from *ab initio* CEMC simulations on various DRX compositions (including formulae with 5% Mn^{4+}) and at various temperatures. b) *F-Mn(0)* plotted against the fraction of cation sites occupied by Mn in the DRX structure at various simulation temperatures.

Supplementary Note 4: Optimizing DRX synthesis

Solid-state synthesis. For all three DRX compositions of interest, a range of reaction temperatures up to the upper limit of our furnace (1100°C) were tested, as well as short (2h) and long (12h) calcination times. While all compositions could be synthesized phase pure at 1100°C, the minimum temperature needed to obtain a phase-pure DRX was found to be 800°C, 900°C, and 1000°C, for LMT53, LMT62, and LMT81, respectively, as shown in Figure S6 below. At those minimal temperatures, a 2h calcination step was sufficient to form the DRX phase.

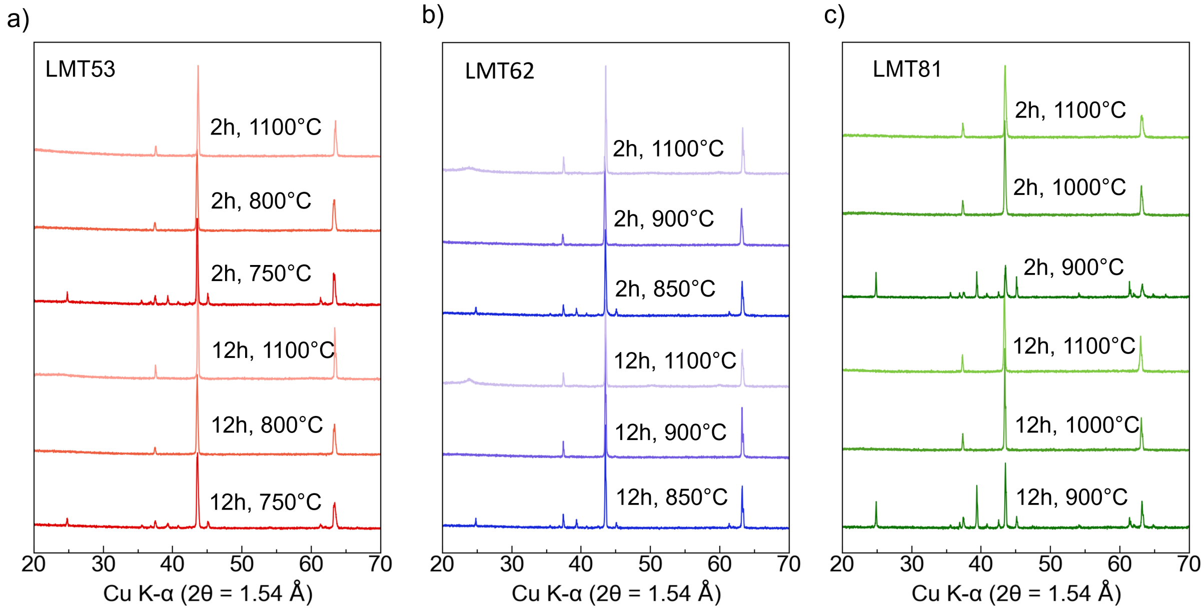

Figure S6: XRD patterns collected on LMT53, LMT62, and LMT81 samples prepared via solid-state synthesis using various sintering temperatures and times.

The Li and F contents in the DRX phase, and the fraction of Li- and F-containing impurities, were obtained using the hybrid experimental-computational method described in Supplementary Notes 1 and 2 for all DRX samples sintered at the minimum temperature needed to obtain a phase-pure DRX (optimal conditions to maximize DRX fluorination), with results shown in Figure S7. Details of the *ab initio* CEMC simulations are described in Supplementary Note 3, and the input parameters used in the simulations are listed in

Table S1. For samples sintered at 1100°C, the Li and F contents shown in Figure S7 correspond to the bulk sample composition (as obtained from ICP and F-ISE measurements) and not to the DRX phase itself. Since those Li and F contents are already drastically reduced compared to samples sintered at lower temperatures, clearly indicating that those conditions are not optimal for DRX fluorination, we did not deem further investigation using ssNMR and CEMC simulations necessary.

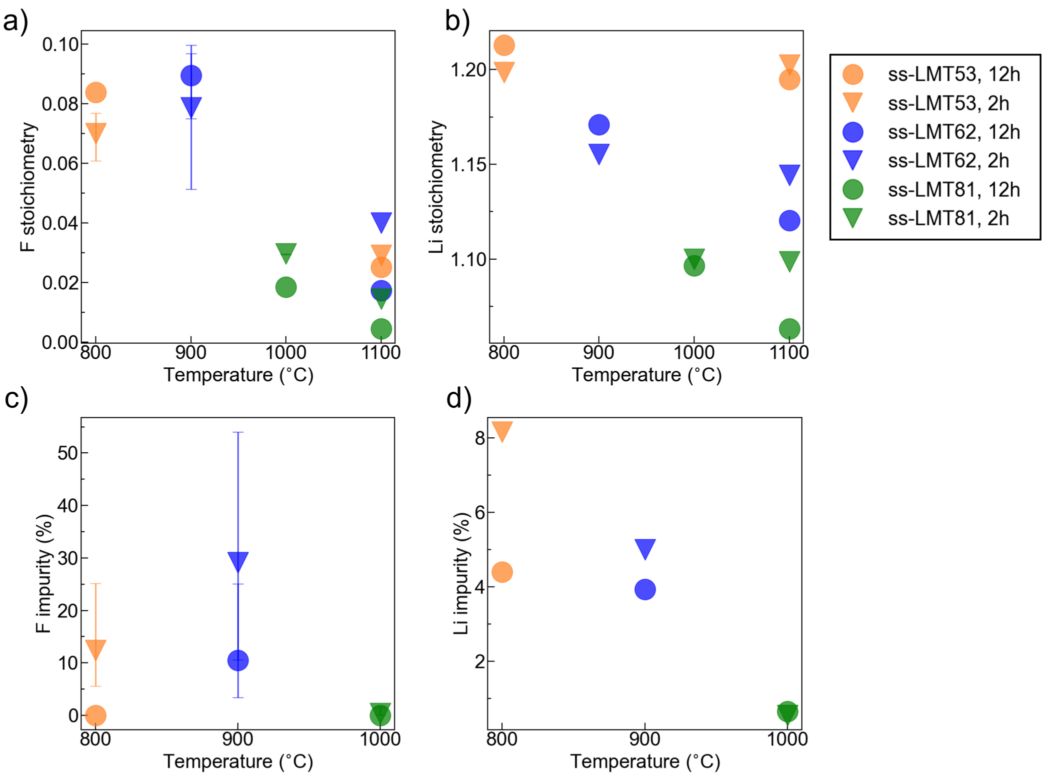

Figure S7: Compositional analysis of LMT53, LMT62, and LMT81 samples prepared via solid-state synthesis using various sintering temperatures and times. (a) Li and (b) F content in the DRX phase for samples synthesized at 800, 900, and 1000°C, and in the bulk sample for those prepared at 1100°C. Amount of (c) Li- and (d) F-containing impurities in samples synthesized at 800, 900, and 1000°C. Error bars in (c) correspond to the upper and lower bounds for the amount of F-containing impurities obtained from ^{19}F ssNMR and assuming a random distribution of species in the DRX structure (see Supplementary Note 2 for details of the analysis).

For all DRX compositions, similar trends were observed regarding the impact of reaction time and temperature on F incorporation into the DRX phase and on the impurity content.

Regardless of whether a 2h or 12h calcination step was used, high reaction temperatures (1100°C) resulted in a drastically reduced amount of F and Li in the DRX phase and sample (Figures S7a-b), which is consistent with increased LiF vaporization. As seen in Figures S7c-d, a longer reaction time of 12h is desired when optimizing for phase purity, as the amount of Li and F impurities in the DRX samples is reduced, particularly for the LMT53 and LMT62 compositions. Interestingly, greater fluorination of the DRX phase can be observed after a 12h calcination step for LMT53 and LMT62, as shown in Figure S7a, indicating that longer reaction times at temperatures of 800 or 900°C favor F incorporation. In contrast, a very low amount of F is able to incorporate the DRX structure for LMT81 (only 1-1.5% fluorination), irrespective of the sintering time.

Overall, the results presented in this section indicate that lower reaction temperatures and longer reaction times result in greater fluorination of the DRX phase, as well as greater volatilization of (at least partially amorphous) F- and Li-based impurities. The DRX stoichiometries of LMT53, LMT62, and LMT81 samples prepared in this way (using the minimum temperature needed to obtain a phase-pure DRX and a 12h calcination step) are listed in Table S2, while the amount of F that is present as LiF or has volatilized during the synthesis is provided (in mols/ pfu of DRX) in Table **??**.

Microwave synthesis. While the precursors used for microwave synthesis were similar to those used for solid-state synthesis, no Li excess was used with this synthesis route. In fact, our recent work on microwave synthesized $Li_{1.2}Mn_{0.4}Ti_{0.4}O_2$ showed that the addition of excess Li results in significant Li-containing impurities in the sample due to a very small amount of Li volatility during the 5 minute heating process.[18]

The only two microwave synthesis parameters that can be controlled with our current setup are reaction time and microwave power. As the reaction temperature depends on both of these factors, the reaction time was held constant at 5 minutes to ensure the formation of a phase-pure product while avoiding melting of the precursor pellet, and only the microwave

power was varied. Two extremes of microwave power were chosen: the maximum available power of 1200W, and the minimum power needed to consistently produce a phase-pure DRX within 5 minutes, corresponding to 720W, 600W, and 600W for mw-LMT53, mw-LMT62, and mw-LMT81, respectively (Figure S8). Although the lower power needed to synthesize higher Mn content DRX may seem counter-intuitive (considering that the solid-state synthesis results indicated that higher temperatures were needed to obtain those systems), we note that Mn-rich oxides appear to couple more strongly to the microwaves, resulting in more effective heating as evidenced by the fact that the mw-LMT81 pellet glows even at low microwave powers.

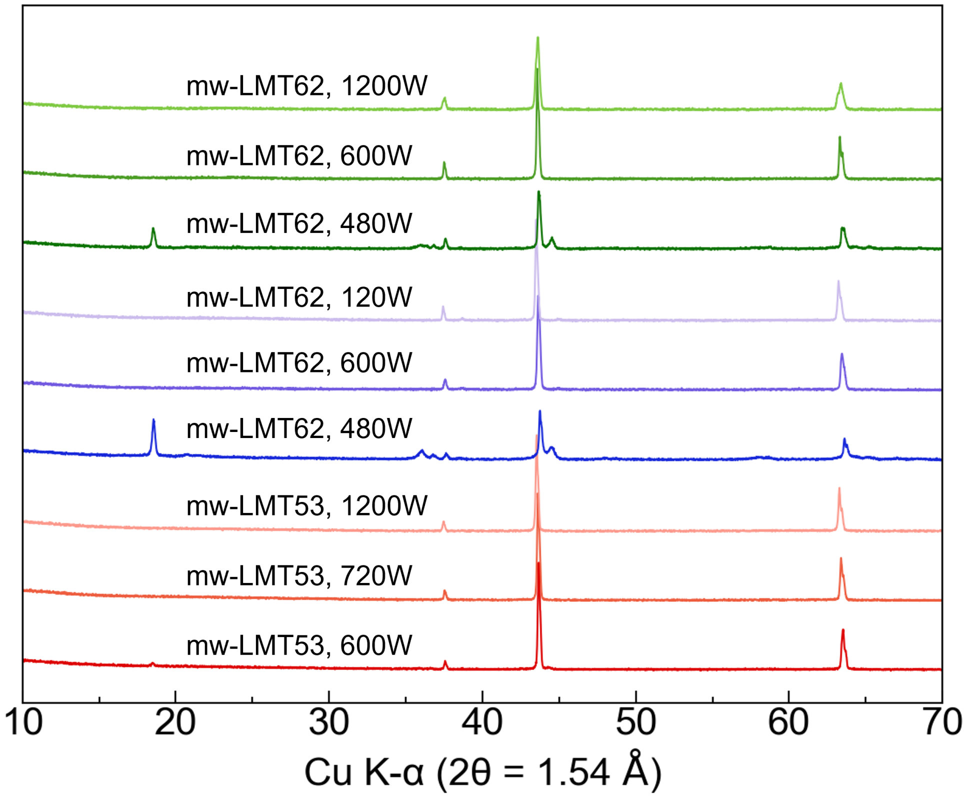

Figure S8: XRD patterns collected on LMT53, LMT62, and LMT81 samples prepared via microwave synthesis using various microwave powers and a 5 minute reaction time.

The Li and F contents in the DRX phase, and the fraction of Li- and F-containing impurities, were obtained using the hybrid experimental-computational method described in

Supplementary Notes 1 and 2 for all microwave-synthesized samples, with results shown in Figure S9. For all DRX compositions of interest, higher microwave powers reduce the F and Li contents in the DRX phase (Figure S9a-b), mirroring results from the high temperature (1100°C) solid-state syntheses. Thus, while the microwave reaction occurs within a few minutes, Li and F volatilization still occurs, likely due to the very high temperatures reached with microwave heating. In fact, the crucible temperature measured a few seconds after the reaction completed was at 1200°C, suggesting that even higher temperatures are reached during microwave irradiation. Figure S9c-d shows the impact of microwave power on Li- and F-containing impurities, where for all samples, no clear trend between microwave power and impurity content is observed.

Overall, the results presented in this section indicate that the highest level of DRX fluorination is obtained when using the minimum microwave power required to form the DRX phase at 5 minute reaction time. The DRX stoichiometries of samples prepared in this way are listed in Table S2, while the amount of F that is present as LiF or has volatilized during the synthesis is provided (in mols/ pfu of DRX) in Table S3.

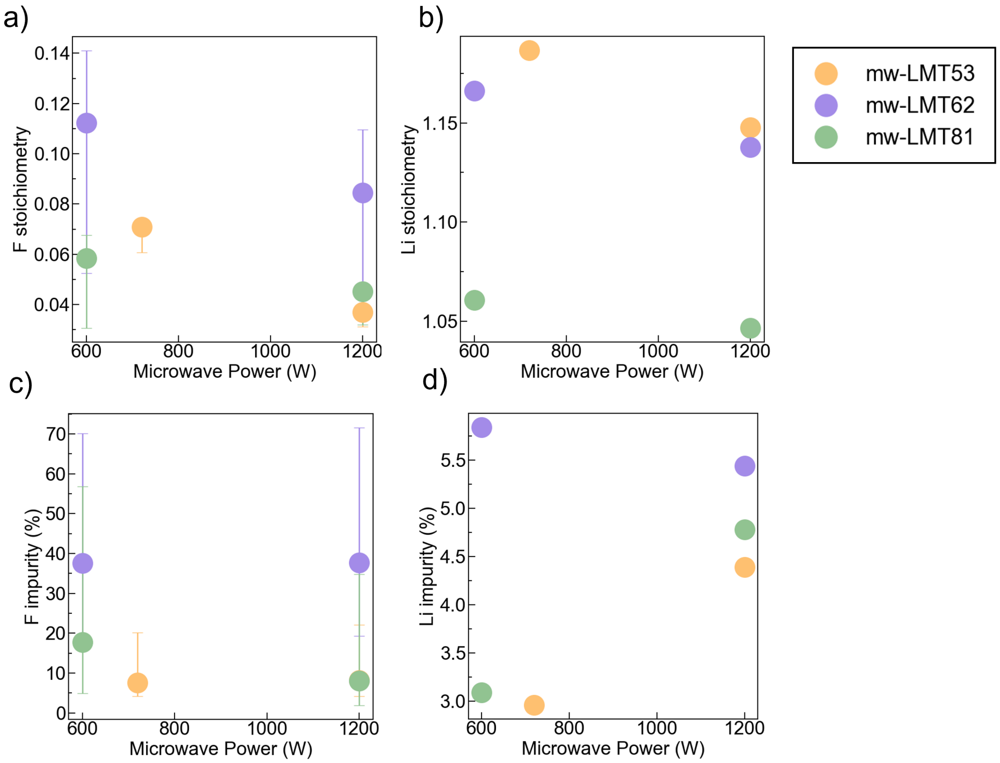

Figure S9: Compositional analysis of LMT53, LMT62, and LMT81 samples prepared via microwave synthesis using various microwave powers and a 5 minute reaction time. (a) Li and (b) F content in the DRX phase, and (c-d) amount of (c) Li- and (d) F-containing impurities in the as-prepared samples. Error bars in (c) correspond to the upper and lower bounds for the amount of F-containing impurities obtained from ^{19}F ssNMR and assuming a random distribution of species in the DRX structure (see Supplementary Note 2 for details of the analysis).

Table S2: DRX stochiometries of all optimized microwave and solid-state synthesized LMT53, LMT62, and LMT81 samples. While the listed F content is the true F stoichiometry in the DRX phase obtained by combining F-ISE, ^{19}F ssNMR and *ab initio* CEMC results, F_{min} and F_{max} correspond to the minimum and maximum possible DRX F contents obtained from ^{19}F ssNMR and assuming a random distribution of species in the DRX structure (see Supplementary Note 2 for details of the analysis). Li_{bulk} corresponds to the total Li content in the DRX sample including impurities, as measured by ICP. The amount of Li impurity (in mol %) refers the molar fraction of Li present as diamagnetic species in the sample.

Sample	Li	Mn	Ti	F	F_{min}	F_{max}	Mn:Ti ratio	Li_{bulk}	Li impurity (mol %)
ss-LMT53, #1	1.21	0.496	0.294	0.088	0.088	0.088	5:2.96	1.28	5.1
ss-LMT53, #2	1.21	0.499	0.294	0.083	0.083	0.083	5:2.95	1.28	5.5
ss-LMT53, #3	1.21	0.495	0.292	0.084	0.084	0.084	5:2.95	1.27	4.4
ss-LMT62, #1	1.16	0.633	0.206	0.085	0.069	0.093	6:1.95	1.21	4.1
ss-LMT62, #2	1.18	0.618	0.204	0.091	0.068	0.105	6:1.98	1.25	5.6
ss-LMT62, #3	1.17	0.625	0.204	0.089	0.075	0.097	6:1.96	1.22	3.9
ss-LMT81, #1	1.09	0.809	0.100	0.016	0.016	0.016	8:0.99	1.10	1.2
ss-LMT81, #2	1.09	0.808	0.099	0.014	0.014	0.014	8:0.98	1.10	1.1
ss-LMT81, #3	1.10	0.804	0.100	0.018	0.018	0.018	8:0.99	1.10	0.7
mw-LMT53, #1	1.19	0.512	0.302	0.071	0.061	0.073	5:2.95	1.22	3.0
mw-LMT53, #2	1.18	0.517	0.304	0.058	0.045	0.061	5:2.93	1.22	3.2
mw-LMT53, #3	1.18	0.517	0.304	0.061	0.046	0.065	5:2.94	1.20	2.0
mw-LMT62, #1	1.17	0.629	0.205	0.112	0.052	0.141	6:1.96	1.24	5.8
mw-LMT62, #2	1.17	0.627	0.206	0.071	0.027	0.101	6:1.97	1.23	5.2
mw-LMT62, #3	1.17	0.622	0.207	0.060	0.021	0.089	6:2.00	1.24	5.8
mw-LMT81, #1	1.06	0.837	0.102	0.058	0.031	0.068	8:0.98	1.09	3.1
mw-LMT81, #2	1.08	0.819	0.102	0.056	0.024	0.070	8:0.99	1.11	2.4
mw-LMT81, #3	1.08	0.823	0.101	0.056	0.026	0.067	8:0.99	1.10	2.1

Table S3: Quantification of F species that are not incorporated into the DRX structure for LMT53, LMT62, and LMT81 samples obtained at the lowest possible sintering temperature (solid-state) or lowest possible microwave power to form the DRX phase. The hybrid experimental-computational analysis was carried out on triplicate samples for improved statistics. The amount of F impurity (in mol %) refers the molar fraction of F present as LiF in the as-prepared sample. The amount of F impurity and of F volatilized during the synthesis (in mols/ mol of DRX) are also obtained.

Sample	F impurity (mol %)	F impurity (mols/ mol DRX)	F volatilized (mols/ mol DRX)
ss-LMT53, #1	0	0	0.012
ss-LMT53, #2	0	0	0.017
ss-LMT53, #3	0	0	0.016
ss-LMT62, #1	12	0.012	0.103
ss-LMT62, #2	19	0.021	0.088
ss-LMT62, #3	11	0.011	0.100
ss-LMT81, #1	0	0	0.084
ss-LMT81, #2	0	0	0.086
ss-LMT81, #3	0	0	0.082
mw-LMT53, #1	8	0.006	0.024
mw-LMT53, #2	13	0.009	0.034
mw-LMT53, #3	15	0.011	0.029
mw-LMT62, #1	38	0.066	0.024
mw-LMT62, #2	54	0.081	0.051
mw-LMT62, #3	61	0.088	0.055
mw-LMT81, #1	18	0.013	0.029
mw-LMT81, #2	26	0.020	0.024
mw-LMT81, #3	22	0.016	0.028

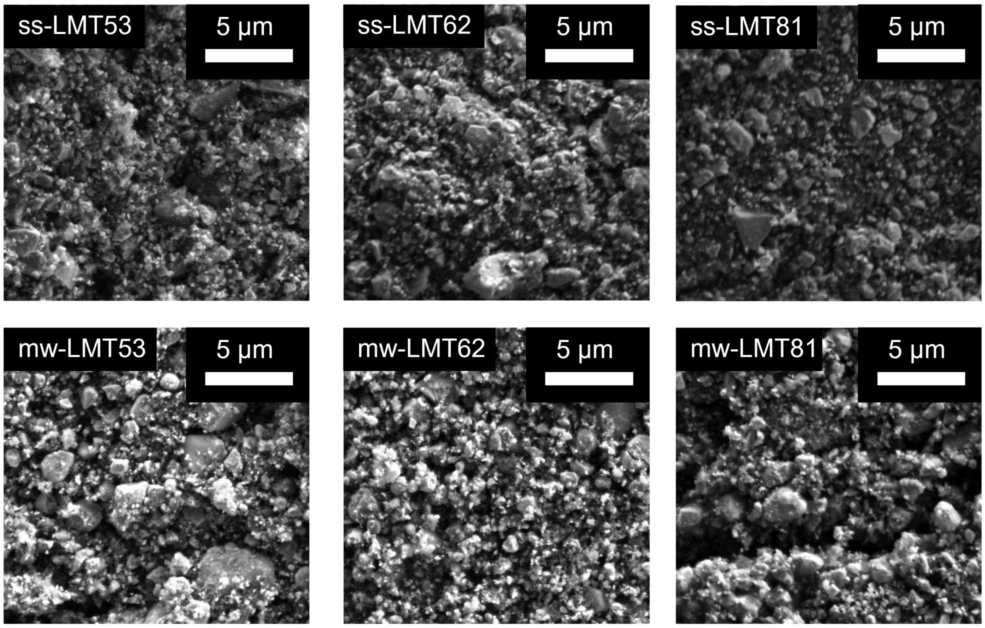

Figure S10: SEM images of solid-state and microwave synthesized LMT53, LMT62, and LMT81, after a ball milling step with Super C65 to downsize and carbon coat active materials.

Additional electrochemical testing of ss- and mw-LMT53, LMT62, and LMT81 DRX cathodes

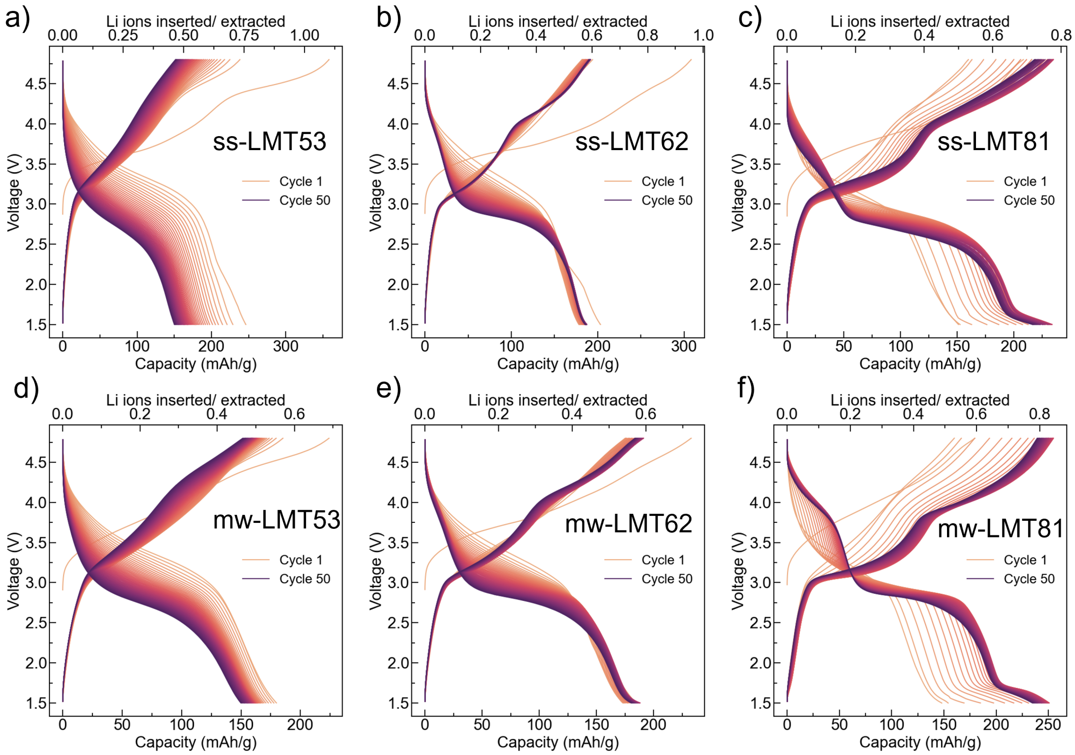

Figure S11: Galvanostatic capacity-voltage plots for solid-state and microwave synthesized LMT53, LMT62, and LMT81.

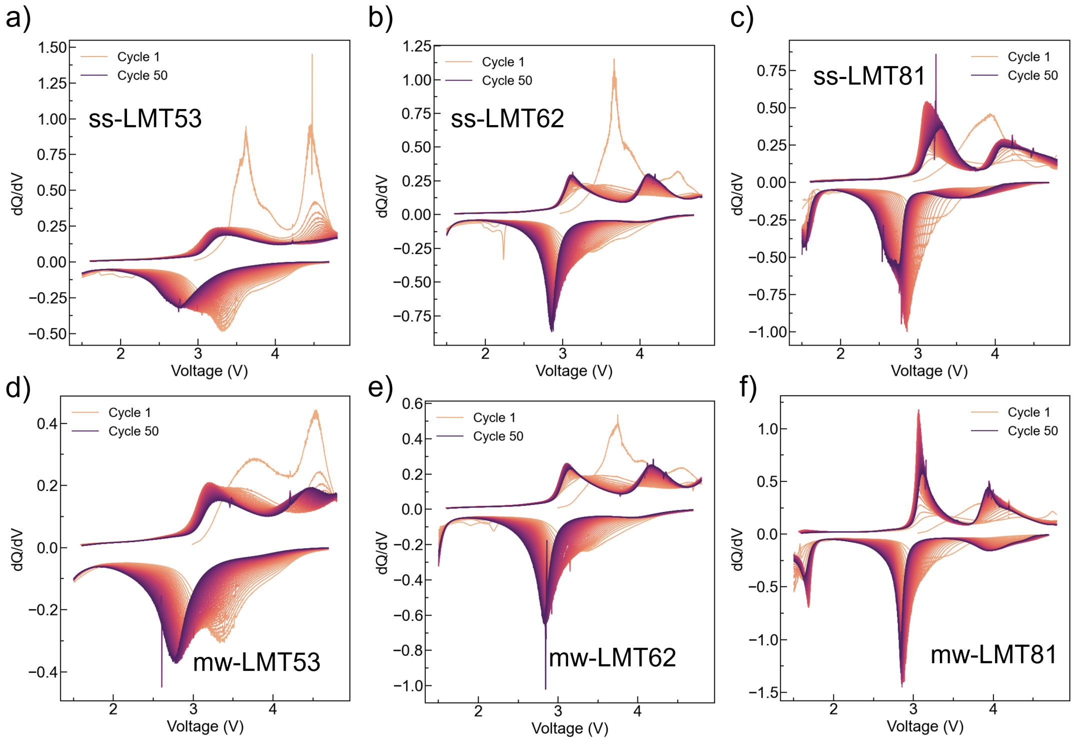

Figure S12: Differential capacity (dQ/dV) plots for solid-state and microwave synthesized LMT53, LMT62, and LMT81.

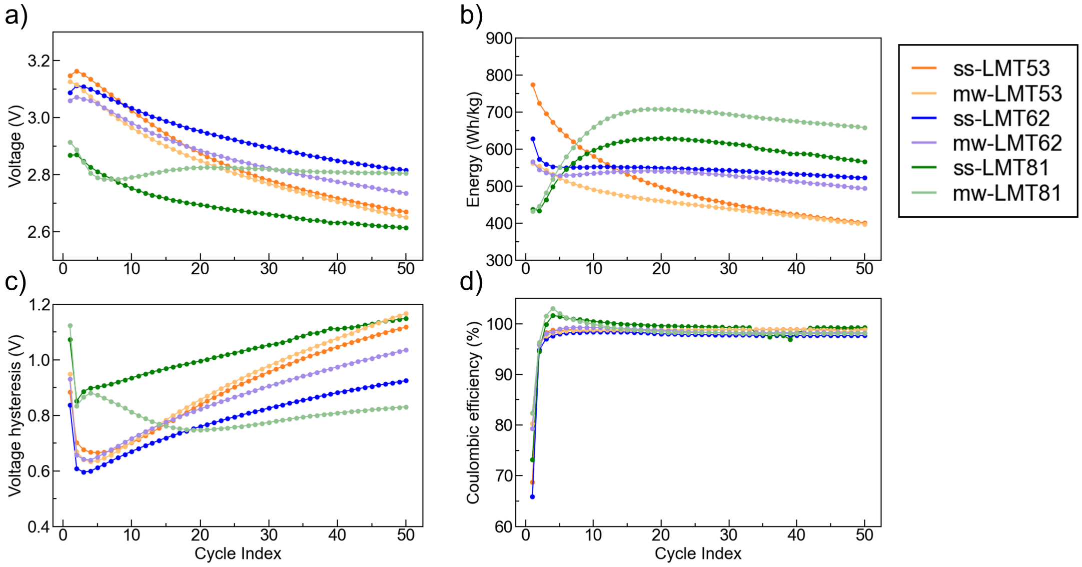

Figure S13: Plots showing the (a) average discharge voltage, (b) energy density, (c) voltage hysteresis (the difference between average charge and discharge voltages), and (d) Coulombic efficiency as a function of cycle index, for solid-state and microwave synthesized LMT53, LMT62, and LMT81.

References

(1) Kresse, G.; Furthmüller, J. Efficiency of ab-initio total energy calculations for metals and semiconductors using a plane-wave basis set. *Computational Materials Science* **1996**, *6*, 15–50.

(2) Furness, J. W.; Kaplan, A. D.; Ning, J.; Perdew, J. P.; Sun, J. Accurate and Numerically Efficient r2SCAN Meta-Generalized Gradient Approximation. *Journal of Physical Chemistry Letters* **2020**, *11*, 8208–8215.

(3) Kingsbury, R.; Gupta, A. S.; Bartel, C. J.; Munro, J. M.; Dwaraknath, S.; Horton, M.; Persson, K. A. Performance comparison of r 2 SCAN and SCAN metaGGA density functionals for solid materials via an automated, high-throughput computational workflow. *Physical Review Materials* **2022**, *6*, 013801.

(4) van de Walle, A. Multicomponent multisublattice alloys, nonconfigurational entropy and other additions to the Alloy Theoretic Automated Toolkit. *Calphad* **2009**, *33*, 266–278.

(5) Zhong, P.; Cai, Z.; Zhang, Y.; Giovine, R.; Ouyang, B.; Zeng, G.; Chen, Y.; Clément, R.; Lun, Z.; Ceder, G. Increasing Capacity in Disordered Rocksalt Cathodes by Mg Doping. *Chemistry of Materials* **2020**, *32*, 10728–10736.

(6) Clément, R. J.; Kitchaev, D.; Lee, J.; Gerbrand Ceder, Short-Range Order and Unusual Modes of Nickel Redox in a Fluorine-Substituted Disordered Rocksalt Oxide Lithium-Ion Cathode. *Chemistry of Materials* **2018**, *30*, 6945–6956.

(7) Huang, L.; Zhong, P.; Ha, Y.; Cai, Z.; Byeon, Y. W.; Huang, T. Y.; Sun, Y.; Xie, F.; Hau, H. M.; Kim, H.; Balasubramanian, M.; McCloskey, B. D.; Yang, W.; Ceder, G. Optimizing Li-Excess Cation-Disordered Rocksalt Cathode Design Through Partial Li Deficiency. *Advanced Energy Materials* **2023**, *13*, 2202345.

(8) Nelson, L. J.; Hart, G. L.; Zhou, F.; Ozoliņš, V. Compressive sensing as a paradigm for building physics models. *Physical Review B* **2013**, *87*, 035125.

(9) Seko, A.; Koyama, Y.; Tanaka, I. Cluster expansion method for multicomponent systems based on optimal selection of structures for density-functional theory calculations. *Physical Review B* **2009**, *80*, 165122.

(10) Zhong, P.; Chen, T.; Barroso-Luque, L.; Xie, F.; Ceder, G. An $\ell_0\ell_2$ -norm regularized regression model for construction of robust cluster expansions in multicomponent systems. *Physical Review B* **2022**, *106*, 024203.

(11) Barroso-Luque, L.; Zhong, P.; Yang, J. H.; Xie, F.; Chen, T.; Ouyang, B.; Ceder, G. Cluster expansions of multicomponent ionic materials: Formalism and methodology. *Physical Review B* **2022**, *106*, 144202.

(12) Barroso-Luque, L.; Yang, J. H.; Xie, F.; Chen, T.; Kam, R. L.; Jadidi, Z.; Zhong, P.; Ceder, G. smol: A Python package for cluster expansions and beyond. *Journal of Open Source Software* **2022**, *7*, 4504.

(13) Ong, S. P.; Richards, W. D.; Jain, A.; Hautier, G.; Kocher, M.; Cholia, S.; Gunter, D.; Chevrier, V. L.; Persson, K. A.; Ceder, G. Python Materials Genomics (pymatgen): A robust, open-source python library for materials analysis. *Computational Materials Science* **2013**, *68*, 314–319.

(14) Coelho, A. TOPAS and TOPAS-Academic: an optimization program integrating computer algebra and crystallographic objects written in C++. *Journal of Applied Crystallography* **2018**, *51*, 210–218.

(15) Giovine, R.; Yoshida, E.; Wu, V. C.; Ji, Y.; Crafton, M. J.; McCloskey, B. D.; Clément, R. J. An Experimental Approach to Assess Fluorine Incorporation into Disordered Rock Salt Oxide Cathodes. *Chemistry of Materials* **2024**, *36*, 3643–3654.

(16) Meyer, B. M.; Leifer, N.; Sakamoto, S.; Greenbaum, S. G.; Grey, C. P. High Field Multinuclear NMR Investigation of the SEI Layer in Lithium Rechargeable Batteries. *Electrochemical and Solid-State Letters* **2005**, *8*, A145.

(17) Ménétrier, M.; Bains, J.; Croguennec, L.; Flambard, A.; Bekaert, E.; Jordy, C.; Biensan, P.; Delmas, C. NMR evidence of LiF coating rather than fluorine substitution in $Li(Ni_{0.425}Mn_{0.425}Co_{0.15})O_2$. *Journal of Solid State Chemistry* **2008**, *181*, 3303–3307.

(18) Wu, V. C.; Evans, H. A.; Giovine, R.; Preefer, M. B.; Ong, J.; Yoshida, E.; Cabelguen, P.-E.; Clément, R. J. Rapid and Energy-Efficient Synthesis of Disordered Rocksalt Cathodes. *Advanced Energy Materials* **2023**, *13*, 2203860.
